# Multi-dimensional fragmentomic assay for ultrasensitive early detection of colorectal advanced adenoma and adenocarcinoma

**DOI:** 10.1186/s13045-021-01189-w

**Published:** 2021-10-26

**Authors:** Xiaoji Ma, Yikuan Chen, Wanxiangfu Tang, Hua Bao, Shaobo Mo, Rui Liu, Shuyu Wu, Hairong Bao, Yaqi Li, Long Zhang, Xue Wu, Sanjun Cai, Yang Shao, Fangqi Liu, Junjie Peng

**Affiliations:** 1 Department of Colorectal Surgery, Fudan University Shanghai Cancer Center, 270 Dong’an Road, Xuhui, Shanghai, 200032 China; 2 Department of Oncology, Shanghai Medical College, Fudan University, Shanghai, 200032 China; 3 Geneseeq Research Institute, Nanjing Geneseeq Technology Inc, Room 1702 Building B Phase I Zhongdan Eco Life Sci Ind Park, Nanjing, 210032 Jiangsu China; 4 Department of Cancer Institute, Fudan University Shanghai Cancer Center, Fudan University, Shanghai, 200032 China

## Abstract

Previous studies on liquid biopsy-based early detection of advanced colorectal adenoma (advCRA) or adenocarcinoma (CRC) were limited by low sensitivity. We performed a prospective study to establish an integrated model using fragmentomic profiles of plasma cell-free DNA (cfDNA) for accurately and cost-effectively detecting early-stage CRC and advCRA. The training cohort enrolled 310 participants, including 149 early-stage CRC patients, 46 advCRA patients and 115 healthy controls. Plasma cfDNA samples were prepared for whole-genome sequencing. An ensemble stacked model differentiating healthy controls from advCRA/early-stage CRC patients was trained using five machine learning models and five cfDNA fragmentomic features based on the training cohort. The model was subsequently validated using an independent test cohort (*N* = 311; including 149 early-stage CRC, 46 advCRA and 116 healthy controls). Our model showed an area under the curve (AUC) of 0.988 for differentiating advCRA/early-stage CRC patients from healthy individuals in an independent test cohort. The model performed even better for identifying early-stage CRC (AUC 0.990) compared to advCRA (AUC 0.982). At 94.8% specificity, the sensitivities for detecting advCRA and early-stage CRC reached 95.7% and 98.0% (0: 94.1%; I: 98.5%), respectively. Promisingly, the detection sensitivity has reached 100% and 97.6% in early-stage CRC patients with negative fecal occult or CEA blood test results, respectively. Finally, our model maintained promising performances (AUC: 0.982, 94.4% sensitivity at 94.8% specificity) even when sequencing depth was down-sampled to 1X. Our integrated predictive model demonstrated an unprecedented detection sensitivity for advCRA and early-stage CRC, shedding light on more accurate noninvasive CRC screening in clinical practice.

**Supplementary Information:**

The online version contains supplementary material available at 10.1186/s13045-021-01189-w.

## To the editor

Recently, researchers have focused on utilizing plasma cell-free DNA (cfDNA), including cfDNA fragmentomic profiles, to develop noninvasive approaches for detecting solid malignancies such as colorectal adenocarcinoma (CRC) [1–6]. But the limited sensitivities of these current detection methods, by the use of either single molecular feature or single algorithm, reduce their potential utilization in clinical practice, while ensembled stacked machine learning approach can improve robustness and accuracy [7, 8]. Herein, we constructed a multi-dimensional ensembled stacked machine learning approach, employing five different base models on five optimized fragmentation features, to provide an ultrasensitive and cost-effective model for detecting early-stage CRC and advanced adenoma (advCRA).

**Fig. 1:**
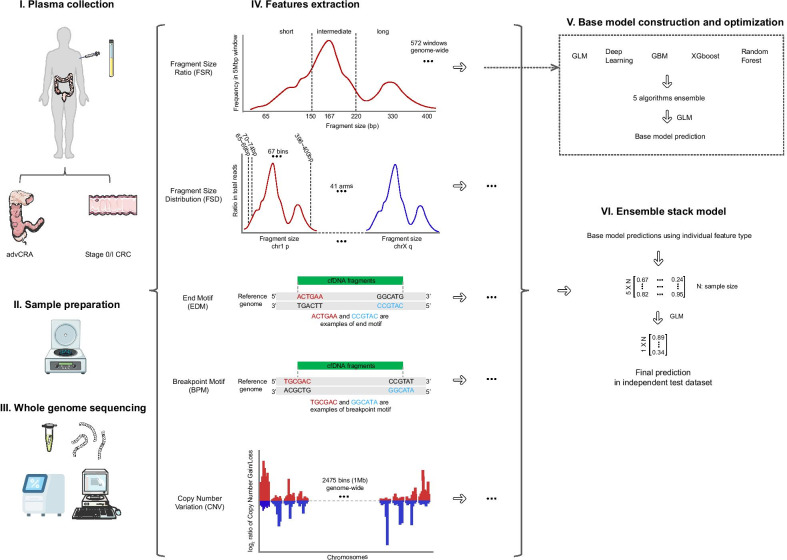
Schematic illustration of study design. Plasma samples were collected from patients with advanced colorectal adenoma (advCRA) or early-stage (stage 0/I) adenocarcinoma (CRC), as well as healthy controls. The cfDNA was then extracted from the participant’s plasma sample and subject to whole-genome sequencing. Five different feature types, including Fragment Size Ratio (FSR), Fragment Size Distribution (FSD), EnD Motif (EDM), BreakPoint Motif (BPM) and Copy Number Variation (CNV), were calculated using mapped sequencing reads. For each feature type, a base model was constructed based on the ensemble learning of five algorithm, GLM, GBM, random forest, deep learning and Xgboost. The base model predictions were then ensembled into a large matrix, which was subsequently used by a GLM algorithm to train the final ensemble stack model

**Fig. 2:**
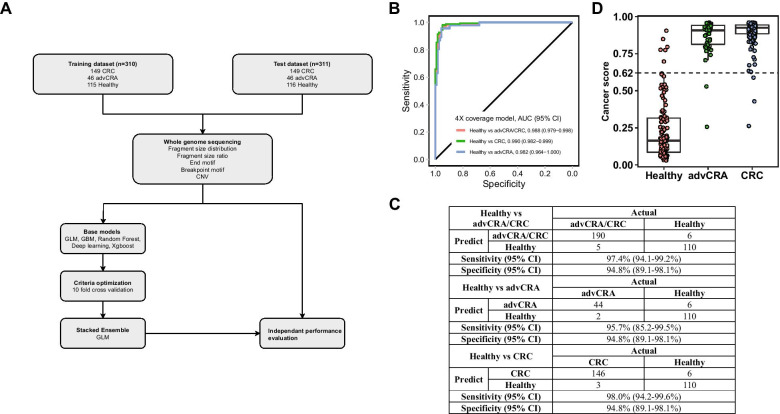
Evaluation of ensemble stacked machine learning model.** A** Graphical representation of datasets composition. The training cohort (*N* = 310) included 149 early-stage CRC patients, 46 advCRA patients and 115 healthy controls and was used to train the stacked ensemble model. The test cohort (*N* = 311), which included 149 early-stage CRC patients, 46 advCRA patients and 115 healthy controls, was independently used to evaluate model performances. **B** ROC curves evaluating the overall performance of the predictive model, which was constructed using 4 X coverage WGS data, in distinguishing advCRA/early-stage CRC patients from healthy controls in the test cohort. **C** Table evaluating model performances in the test dataset. **D** Boxplots illustrating cancer score distribution in the healthy, advCRA and early-stage CRC groups in the test cohort based on the 4 X overage model. The 95% specificity cutoff for cancer score was 0.62 as shown by the dotted line

In this study, 149 early-stage colorectal adenocarcinoma (CRC) patients, 46 advCRA patients and 115 healthy volunteers were recruited in the training cohort from a single center, which was used to train the machine learning models (Figs. 1, 2A). To eliminate the potential impact on the predictive power by the different coverages and maximize affordability, WGS data were down-sampled to 4X coverage, unless otherwise noted. The test cohort (*N* = 311), which consisted of 149 early-stage CRC, 46 advCRA patients and 116 healthy controls, was used to evaluate model performances. ROC curves were constructed using five individual features including Fragment Size Ratio (FSR), Fragment Size Distribution (FSD), EnD Motif (EDM), BreakPoint Motif (BPM) and Copy Number Variation (CNV), as well as the DELFI fragment size pattern [1] and the 4-bp end-motif pattern by Jiang et al. [2], to demonstrate the advantage of using a multi-dimensional ensembled stacked machine learning model approach, as well as adapting existing fragmentation features [7]. Detailed methodology is described in supplementary methods section (Additional file 1).

The ensembled stacked model had a higher AUC (0.988) than base models using any individual feature (AUC range 0.881–0.981), validating the multi-dimensional ensembled stacked approach (Additional file 1: Fig. S1). A similar pattern was observed as the ensembled stacked model had the highest sensitivity for detecting advCRA/early-stage CRC (97.4%, 95% CI 94.1–99.2%) compared to all base models (sensitivity range 57.4–89.2%) at 94.8% specificity (95% CI 89.1–98.1%) (95% CI 89.1–98.1%) (Additional file 1: Fig. S1, Table S1). Additionally, our adaptation to the existing fragmentation features was justified by showing better performances than the original features: the adapted 6-bp EDM feature showed higher AUC (0.981, 95% CI 0.969–0.993) than the original 4-bp end-motif feature (0.969, 95% CI 0.953–0.985), while models using FSR or FSD both had higher AUC (0.881, 95% CI 0.843–0.919; 0.892, 95% CI 0.855–0.930) than the original DELFI fragment pattern (Additional file 1: Fig. S1).

The stacked model showed better AUC while differentiating early-stage CRC (0.990, 95% CI 0.981–0.998) than advCRA (0.983, 95% CI 0.968–0.999) (Fig. 2B). Similarly, the model showed excellent sensitivities for detecting both advCRA (95.7%, 95% CI 85.2–99.5%) and early-stage CRC (98.0%, 95% CI 94.2–99.6%) at the 94.8% specificity (95% CI 89.1–98.1%) (Fig. 2D). The advCRAs more closely resembled early-stage CRCs than healthy controls (Fig. 2C), which was further validated by two additional models (Additional file 1: Fig. S2A, S2B). The current gold standard colonoscopy can be used to histopathologically distinguish advCRA and early-stage CRC following our model’s predictions.

We then constructed an ensembled stacked model using the raw depth NGS data (4.7–24.04X, median 9.75X), still showing great performances an identical AUC of 0.988 (95% CI 0.979–0.997) (Additional file 1: Fig. S3, Table S2). A limit of detection analysis was performed by further down-sampling the 4X coverage WGS data to 3X, 2X, 1X and 0.5X. The down-sampled data was then used to evaluate the 4X model. The AUCs showed a gradual decrease during the down-sampling process (0.988, 0.987, 0.985, 0.982 and 0.977 for 4X, 3X, 2X, 1X and 0.5X data, respectively) (Additional file 1: Fig. S4A).

In summary, our multi-dimensional ensembled stacked model, which uses plasma cfDNA WGS data, showed great potential for accurate noninvasive colorectal cancer screening prior to the current gold standard colonoscopy in clinical practice by demonstrating an unparalleled high sensitivity in detecting early-stage CRC as well as advCRA. However, this study was limited by several factors, namely the relatively small cohort size. The small number of healthy controls within the test cohort can impact the model performance, likely resulting in an underestimation of sensitivity. A multicenter, large-scale prospective study is needed to validate the clinical value of our methods further.

## Supplementary Information

**Additional file 1: Supplementary methods. Supplementary Results. Supplementary Figures.** **Figure S1.** Evaluation of base model using individual features. **Figure S2.** Evaluation of models distinguishing advCRA from early-stage CRC or healthy controls. **Figure S3.** Evaluation of model constructed using raw coverage WGS data. **Figure S4.** Evaluation of a multi-dimensional model detecting advCRA/early-stage CRC. **Figure S5.** Evaluation of age and gender matched groups in the test cohort. **Figure S6.** Evaluation of model using 10-fold cross-validation score of the training cohort. **Supplementary Tables. Table S1.** Performances evaluation of base models using different features. **Table S2.** Evaluating performances of model constructed by raw depth data in the test dataset. **Table S3.** Participant demographics and baseline characteristics. **Table S4.** Clinical information of the colorectal advanced adenoma (advCRA) and Adenocarcinoma (CRC) patients.

## Data Availability

The datasets used and/or analyzed during the current study are available from the corresponding author on reasonable request.

## Abbreviations

advCRA: Advanced colorectal adenoma
CRC: Colorectal adenocarcinoma
cfDNA: Cell-free DNA
WGS: Whole-Genome Sequencing
AUC: Area Under the Curve
CNV: Copy number variation
DELFI: DNA EvaLuation of Fragments for early Interception
FSR: Fragment Size Ratio
FSD: Fragment Size Distribution
EDM: EnD Motif
BPM: BreakPoint Motif
