# Supplementary material for: Multi-dimensional fragmentomic assay for ultrasensitive early detection of colorectal advanced adenoma and adenocarcinoma
Source: J Hematol Oncol. 2021 Oct 26;14:175. doi: 10.1186/s13045-021-01189-w (PMC8549237; doi:10.1186/s13045-021-01189-w)
Supplement: Supplementary file 1 — Additional file 1: Supplementary methods. Supplementary Results. Supplementary Figures. Figure S1. Evaluation of base model using individual features. Figure S2. Evaluation of models distinguishing advCRA from early-stage CRC or healthy controls. Figure S3. Evaluation of model constructed using raw coverage WGS data. Figure S4. Evaluation of a multi-dimensional model detecting advCRA/early-stage CRC. Figure S5. Evaluation of age and gender matched groups in the test cohort. Figure S6. Evaluation of model using 10-fold cross-validation score of the training cohort. Supplementary Tables. Table S1. Performances evaluation of base models using different features. Table S2. Evaluating performances of model constructed by raw depth data in the test dataset. Table S3. Participant demographics and baseline characteristics. Table S4. Clinical information of the colorectal advanced adenoma (advCRA) and Adenocarcinoma (CRC) patients. [file 13045_2021_1189_MOESM1_ESM.pdf]

Methods

Participants enrollment and cohort design

This study enrolled three types of participants, including early-stage CRC patients, advanced colorectal adenoma (advCRA) patients and healthy volunteers. Participants, who were 18 years and older, were eligible to enroll. Advanced colorectal adenoma was defined as an adenoma with a diameter >= 10 mm, a villous adenoma (i.e., at least 25 percent villous) or high-grade dysplasia, according to the National Comprehensive Cancer Network (NCCN) colorectal cancer screening guideline (version 2.2021) [1]. The early-stage CRC patients included stage 0 (carcinoma *in situ*) and stage I according to the American Joint Committee on Cancer (AJCC) TNM system (8th edition) [2].

The inclusion criteria for advCRA and CRC group include: A) Able to provide sufficient and qualified blood samples for study tests; B) Able to provide a written informed consent; C) Histopathological diagnosis with advCRA or stage I CRC. The exclusion criteria for advCRA and CRC group include : A) Female participants during pregnancy or lactation; B) Currently diagnosed with other types of tumor or any cancer history; C) Patients diagnosed with hereditary colorectal cancer; D) No prior or undergoing cancer treatment, including surgery, chemotherapy, radiotherapy, targeted therapy and immunotherapy, prior to blood draw; E) Ongoing fever or recipient of anti-inflammation therapy within 14 days prior to study blood draw; F) Recipient of blood transfusion within 30 days prior to study blood draw; G) Recipient of organ transplant or prior non-autologous (allogeneic) bone marrow or stem cell transplant; H) Poor health condition and not suitable for blood draw; I) Any other disease/condition deemed not suitable for study enrollment by researcher.

The inclusion criteria for healthy volunteers include: A) Able to provide sufficient and qualified blood samples for study tests; B) Able to provide a written informed consent; C) No cancer related symptoms within 30 days prior to enrollment; D) No history of cancer diagnosis and treatment. The exclusion criteria include: A) Female participants during pregnancy or lactation; B) Ongoing fever or recipient of anti-inflammation therapy within 14 days prior to study blood draw; C) Recipient of blood transfusion within 30 days prior to study blood draw; D) Recipient of organ transplant or prior non-autologous (allogeneic) bone marrow or stem cell transplant;

The cohort sizes were calculated using a R package MKmisc (version 1.8), which was developed to determine sample sizes in binary classification tests [3, 4]. Based on the calculation, ~203 advCRA/early-stage CRC patients were enough for testing the desired 95% sensitivity within a margin of error of 5% (significance level = 0.05, power of test = 0.8). ~117 non-cancer controls were needed for testing the desired 95% specificity within a margin of error of 6% (significance level = 0.05, power of test = 0.8). A total of 310 participants were recruited in the training cohort, including 149 early-stage CRC patients, 46 advCRA patients and 115 healthy controls. While the test cohort enrolled 311 participants, including 149 early-stage CRC patients, 46 advCRA patients and 115 healthy controls. The participants were assigned to an independent training and an independent testing cohort at a 1:1 ratio by the personnel who were collecting the clinical information. The team involved in cohort design was blinded to the sequencing, feature extraction, model construction and validation process. The training cohort was used to train the multi-omics machine learning model, while the test cohort was used to evaluate model performances. The early-stage CRC patients and advCRA patients were enrolled from Department of Colorectal Surgery, Fudan University Shanghai Cancer Center (FUSCC), Shanghai, and the healthy volunteers who had no history of major diseases were recruited in physical examination centers. This study was

approved by the Shanghai Cancer Center Institutional Review Board (SCCIRB), which is the ethnic committee of FUSCC, and in accordance with international standards of good clinical practice. Written informed consents were provided by all participants.

WGS and bioinformatics pipelines

Available peripheral blood samples collected from participants in this study underwent cfDNA extraction followed by WGS. For the advCRA and early-stage CRC patients, the peripheral blood samples were collected after diagnosis and before any treatment. The plasma samples were centrifuged at 16000x for 10 mins to remove cell debris, followed by cfDNA extraction using QIAamp Circulating Nucleic Acid Kit (Qiagen) according to the manufacturer's protocols. The cfDNA concentrations in these plasma samples were examined with Qubit dsDNA HS Assay Kit (Thermo Fisher Scientific) as per manufacturer guidelines.

WGS libraries were constructed using the KAPA Hyper Prep Kit (KAPA Biosystems) according to the manufacturer's protocol. In brief, 5-10 ng of cfDNA per sample was subjected to end-repairing, A-tailing, and ligation with adapters sequentially. The libraries were quantified with the KAPA SYBR FAST qPCR Master Mix (KAPA Biosystems) and underwent paired-end sequencing on NovaSeq platforms (Illumina) according to the manufacturer's instructions.

Raw sequencing data were trimmed by Trimmomatic as part of the quality control (QC) protocol [5]. The qualified reads were then mapped onto the human reference genome (GRCh37/UCSC hg19) using the sequence aligner bwa [6] after PCR duplicates removal by Picard toolkit (http://broadinstitute.github.io/picard/). The final coverage depths for these samples were ranging from 4.7X to 24.04X (median coverage 9.75X).

Multi-dimensional featurization and machine learning model construction

We then used the processed WGS data to extract different features, including the fragmentation, motif and copy number variation profiles, for constructing a multi-dimensional machine learning model detecting advCRA and early-stage CRC.

Two new fragment size profiles, Fragment Size Ratio (FSR) and Fragment Size Distribution (FSD), were adapted from the DELFI fragment size profile in order to improve the predicting ability[7]. The fragments sizes were used to construct these fragmentation profiles using in-house scripts (Fig. 1). The FSR, similar to the original fragmentation profile reported by DELFI [7], was generated using the short/intermediate/long fragments ratios except using different cutoffs: the short, intermediate and long fragments were defined as 65-150bp, 151-220bp and 221-400bp, according to the overall fragment lengths profile in our cohorts. The new cutoff allowed the inclusion larger fragment size regions, as well as additional regions between 221-400bp which provided extra information for distinguishing cancer and non-cancer groups, compared to the reported 100-150bp/151-220bp short/long regions, therefore theoretically improving model performances. For each sample , the ratio between short and long fragments were examined in 5Mb bins, forming 572 bins genome-wide. The FSD examined fragment length patterns at a high resolution by grouping cfDNA fragments into length bins of 5bp ranging from 65bp and 400bp and calculating the ratio of fragments in each bin at arm level for each chromosome. A total of 41 chromosome arms were examined. The high-resolution pattern at each chromosome level should reveal more differences between cancer and non-cancer groups.

The motif profile was constructed by aligning the fragments back onto the human reference genome hg19. Two types of motifs, including the EnD Motif (EDM) and BreakPoint Motif (BPM), were extracted from the processed WGS data using in-house scripts (Fig. 1). The EDM was

adapted from the end motif reported initially by Jiang et al. [8] by extending the 4bp 5' end motif to 6bp, which examined the motif pattern at a higher resolution by increasing the 256 (4^4) patterns into 4096 (4^6). The BPM examined frequencies of the 6bp motif at the 5' breakpoints on the human reference genome hg19, which extended 3bp to each direction. The BPM, in theory, carries more information as it includes the pattern at the fragment origin sites within the genome, which may increase its' predictive ability.

The Copy Number Variation profile was calculated using ichorCNA according to the report by Wan et al. [9]. For each sample, the genome was divided into 1Mb bins, forming a total of 2475 bins. The depth for each bin was then used by a Hidden Markov Model (HMM) to compare against software baseline, and the $\log_2$ ratio for each bin was calculated.

The total 310 participants in the training cohort, as shown in Fig. 1&2, were categorized into cancer group (149 early-stage CRC and 46 advCRA) and healthy control group (115 healthy controls). In total, five base models were constructed for the five feature types, including FSR, FSD, EDM, BPM and CNV, using the training cohort (Fig. 1). Each of the five base models implemented five different algorithms, including Generalized Linear Model (GLM), Gradient Boosting Machine (GBM), Random Forest, Deep Learning and XGBoost. Healthy control and advCRA/early-stage CRC patient samples from the training cohort were used by each algorithm to train the given classifier and to generate the corresponding cancer score for each sample. The cancer score ranged from 0 to 1, while a higher score representing a higher probability for cancer. The cancer scores from all five algorithms were ensembled into a matrix, which was subsequently used by a second layer GLM algorithm to create the base model (Fig. 1). 10-fold cross-validation was performed for each of these five base models for criteria optimization.

The optimized base models were then used to create the final multi-dimensional model through ensembled stack machine learning by a GLM algorithm, which has been shown to improve a decision systems' robustness and accuracy in many other disciplines [10, 11]. Similarly, the cancer scores predicted by each of the five base models using solely training cohort samples were ensembled into a matrix, which was used as input for the GLM algorithm. Finally, the test cohort was used to evaluate the performance of our multi-dimensional ensembled machine learning model at 95% specificity.

To eliminate the potential impact on the predictive power by the different coverages among the WGS data, we down-sampled the coverages to a 4X, ensuring the inclusion of all samples.

Statistical Analysis

The receiver operating characteristic (ROC) curves were constructed using the pROC package (v. 1.17.0.1). Based on true-positive (TP), true-negative (TN), false-positive (FP), and false-negative (FN) of cancer prediction, the sensitivity [TP/(TP+FN)], specificity [TN/(TN+FP)], positive (PPV) [TP/(TP+FP)] and negative predictive (NPV) [TN/(TN+FN)] values, accuracy [(TP+TN)/(TP+FP+TN+FN)] as well as the corresponding 95% confidence intervals, were calculated using the epiR package (v 2.0.19). Propensity score matching analysis of age and gender within the test cohort was performed using the MatchIt package (4 4.2.0). All statistical analyses were performed in R (v.3.6.3).

Supplementary results

The mean age for the early-stage CRC patients was 61.1 years in the training cohort and 62.1 years in the test cohort, compared to the mean age of 58.7 years (training cohort) and 58.0 years (test cohort) for advCRA patients and 45.5 years (training cohort) and 46.4 years (test cohort) for healthy controls, as shown in Table S3, S4. The majority of early-stage CRC patients enrolled in this study belonged to stage I (132, 88.6%), while the rest (17, 11.4%) were stage 0 in both the training and test cohorts (Table S3, S4). As shown in Table S3, S4, 52 early-stage CRC patients had a negative fecal occult blood test (FOBT) in the training cohort, compared to 43 early-stage CRC patients with negative FOBT results in the test cohort. The percentage of negative FOBT reached 43.5% and 45.7% among the advCRA patients in the training and test cohorts, respectively (Table S3, S4). Similarly, as shown in Table S3, S4, the percentages for patients, who had negative cancer embryonic antigen (CEA) or carbohydrate antigen (CA199), was high among both advCRA group (negative CEA: 87.0% and 93.5%, negative CA199: 93.5% and 89.3%) and early-stage CRC group (negative CEA: 89.3% and 83.9%, negative CA199: 92.0% and 91.3%) in the training and test cohorts.

We evaluated the 4X coverage model's ability to detect advCRA and early-stage CRC at different specificity level. As expected, the sensitivities for detecting advCRA (97.8%, 95% CI: 88.5-99.9%), early-stage CRC (98.7%, 95% CI: 95.2-99.8%) and advCRA/early-stage CRC combined (98.5%, 95% CI: 95.6-99.7%) increased when the specificity was lowered to 89.7% (95% CI, 82.6-94.5%) and decreased (80.4% [95% CI, 66.1-90.6%], 91.3% [95% CI, 85.5-95.3%] and 88.7% [95% CI, 83.4-92.8%], for advCRA, early-stage CRC and combined, respectively) at 98.3% specificity (98.3%, 95% CI: 93.9-99.8%) (Table S5).

A decrease in sensitivities for detecting advCRA/early-stage CRC at 89.7% specificity (sensitivity range: 95.4-98.5%) or 94.8% specificity (sensitivity range: 90.8-97.4%) was visualized during the downsampling process, (Fig. S4b, Table S6).

We then focused on our model's performances for the different advCRA and early-stage CRC subgroups. As shown in Table S7, our model performed very well while detecting stage 0 and stage I CRC patients. The sensitivity for detecting the 17 stage 0 CRC patients reached 94.1% (95% CI: 71.3-99.9%), while 130 of the 132 stage I patients were correctly predicted as cancer by our model (sensitivity 98.5%, 95% CI: 94.6-99.8%). Furthermore, our model showed promising detection sensitivities for early-stage CRC patients with negative results for FOBT (100%, 95% CI: 91.8-100%), CEA (97.6%, 95% CI: 93.1-99.5%) or CA199 (97.8%, 95% CI: 93.7-99.5%) (Table S7). Promisingly, as shown in Table S7, our model also demonstrated high sensitivities for detecting KRAS-wt (97.1%, 95% CI: 89.8-99.6%), NRAS-wt (99.1%, 95% CI: 95.2-100.0%) and BRAF-wt (98.2%, 95% CI: 93.8-99.8%) patients.

Similar to the early-stage CRC group, our model also yielded high sensitivity in for advCRA patients with negative FOBT (95.2%, 95% CI: 76.2-99.9%), CEA (95.3%, 95% CI: 84.2-99.4%) or CA199 (95.1%, 95% CI: 83.5-99.4%) results, as shown in Table S7. Overall, our model showed superior sensitivity for detecting advCRA/early-stage CRC patients (97.6%) compared to the traditional FOBT (49.2%) and CEA (13.8%) methods in the test cohort (detail data not shown). In advCRA subgroup, our model showed high sensitivities for detecting different grades of dysplasia (high: 91.3% [95% CI: 72.0-98.9%], low: 100% [95% CI: 84.6-100.0%]) and for pedunculated (92.9%, 95% CI: 66.1-99.8%) or sessile adenoma (96.9%, 95% CI: 83.8-99.9%) (Table S7).

To investigate the potential impact of age or gender inequality between the advCRA/early-stage CRC and the healthy control groups, we have performed a propensity score matching

analysis. A subset consisting of 25 advCRA, 67 early-stage CRC and 72 healthy controls were selected from the test cohort with matching age and gender. As shown in Fig. S5, the age/gender-matched subset still showed identical excellent AUC (0.988, 95% CI: 0.975-1.000) compared to the entire test cohort (0.988, 95% CI: 0.979-0.997).

As shown in Fig. S6, the 10-fold cross-validation in the training cohort reached an AUC of 0.958 (95% CI: 0.938-0.978) while differentiating early-stage CRC/advCRA patients from healthy controls. The AUCs for distinguishing either early-stage CRC or advCRA from healthy control reached 0.955 (95% CI: 0.931-0.978) and 0.969 (95% CI: 0.944-0.994), respectively.

List of abbreviation

advCRA: advanced colorectal adenoma

CRC: colorectal adenocarcinoma

cfDNA: cell-free DNA

WGS: Whole Genome Sequencing

AUC: Area Under the Curve

SEER: Surveillance, Epidemiology, and End Results

NCI: National Cancer Institute

FOBT: fecal occult blood test

CTVE: Computed Tomography virtual endoscopy

NCCN: National Comprehensive Cancer Network

ESMO: European Society for Medical Oncology

mSEPT9: methylated Septin 9

CNV: copy number variation

DELFI: DNA EvaLuation of Fragments for early Interception

AJCC: American Joint Committee on Cancer

FUSCC: Fudan University Shanghai Cancer Center

FSR: Fragment Size Ratio

FSD: Fragment Size Distribution

EDM: EnD Motif

BPM: BreakPoint Motif

HMM: Hidden Markov Model

GLM: Generalized Linear Model

GBM: Gradient Boosting Machine

TP: true-positive

TN: true-negative

FP: false-positive

FN: false-negative

PPV: positive predictive value

NPV: negative predictive value

Method references

1. National Comprehensive Cancer Network. *Colorectal Cancer Screening (Version 2.2021)*. June 14, 2021]; Available from: https://www.nccn.org/professionals/physician_gls/pdf/colorectal_screening.pdf.
2. Amin, M.B., et al., *The Eighth Edition AJCC Cancer Staging Manual: Continuing to build a bridge from a population-based to a more "personalized" approach to cancer staging.* CA Cancer J Clin, 2017. **67**(2): p. 93-99.
3. Kohl, M., *{{MKmisc}: Miscellaneous functions from {M}. {K}ohl},*. 2021, {Matthias Kohl}.
4. Flahault, A., M. Cadilhac, and G. Thomas, *Sample size calculation should be performed for design accuracy in diagnostic test studies.* J Clin Epidemiol, 2005. **58**(8): p. 859-62.
5. Bolger, A.M., M. Lohse, and B. Usadel, *Trimmomatic: a flexible trimmer for Illumina sequence data.* Bioinformatics, 2014. **30**(15): p. 2114-20.
6. Li, H. and R. Durbin, *Fast and accurate short read alignment with Burrows-Wheeler transform.* Bioinformatics, 2009. **25**(14): p. 1754-60.
7. Cristiano, S., et al., *Genome-wide cell-free DNA fragmentation in patients with cancer.* Nature, 2019. **570**(7761): p. 385-389.
8. Jiang, P., et al., *Plasma DNA End-Motif Profiling as a Fragmentomic Marker in Cancer, Pregnancy, and Transplantation.* Cancer Discov, 2020. **10**(5): p. 664-673.
9. Wan, N., et al., *Machine learning enables detection of early-stage colorectal cancer by whole-genome sequencing of plasma cell-free DNA.* BMC Cancer, 2019. **19**(1): p. 832.
10. Zhang, C. and Y. Ma, *Ensemble Machine Learning: Methods and Applications*. 2012: Springer Publishing Company, Incorporated.
11. Kwon, H., J. Park, and Y. Lee, *Stacking Ensemble Technique for Classifying Breast Cancer.* Healthc Inform Res, 2019. **25**(4): p. 283-288.

Supplementary figures

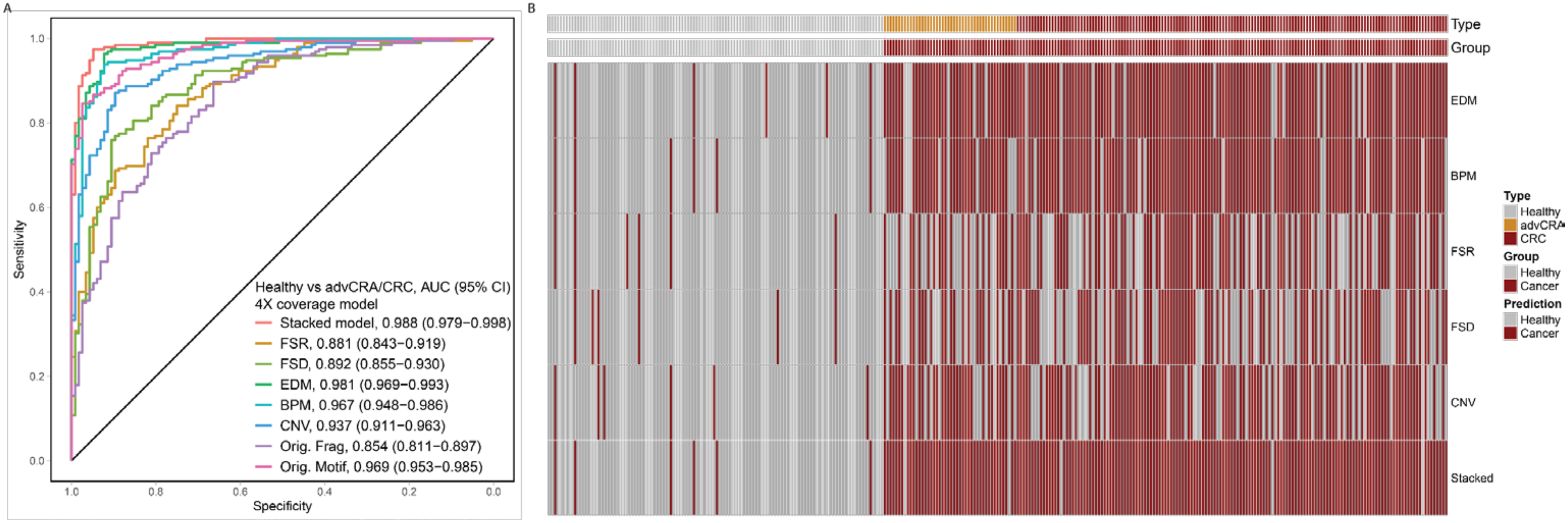

Figure S1. Evaluation of base model using individual features.

A ROC curves of base models using individual features, including FSR, FSD, EDM, BPM and CNV, as well as the two feature type originally reported by Cristiano et al. and Jiang et al. **B** Heatmap showing the predicted status of all 311 sample in the test cohort (149 early-stage CRC, 46 advCRA and 115 healthy) by base models using individual feature type as well as by the ensemble stacked model.

FSR: Fragment Size Ratio; FSD: Fragment Size Distribution: EDM: EnD Motif; BPM: BreakPoint Motif; CNV: Copy Number Variation. Orig. Frag: Original fragmentation; Orig. Motif: Original motif.

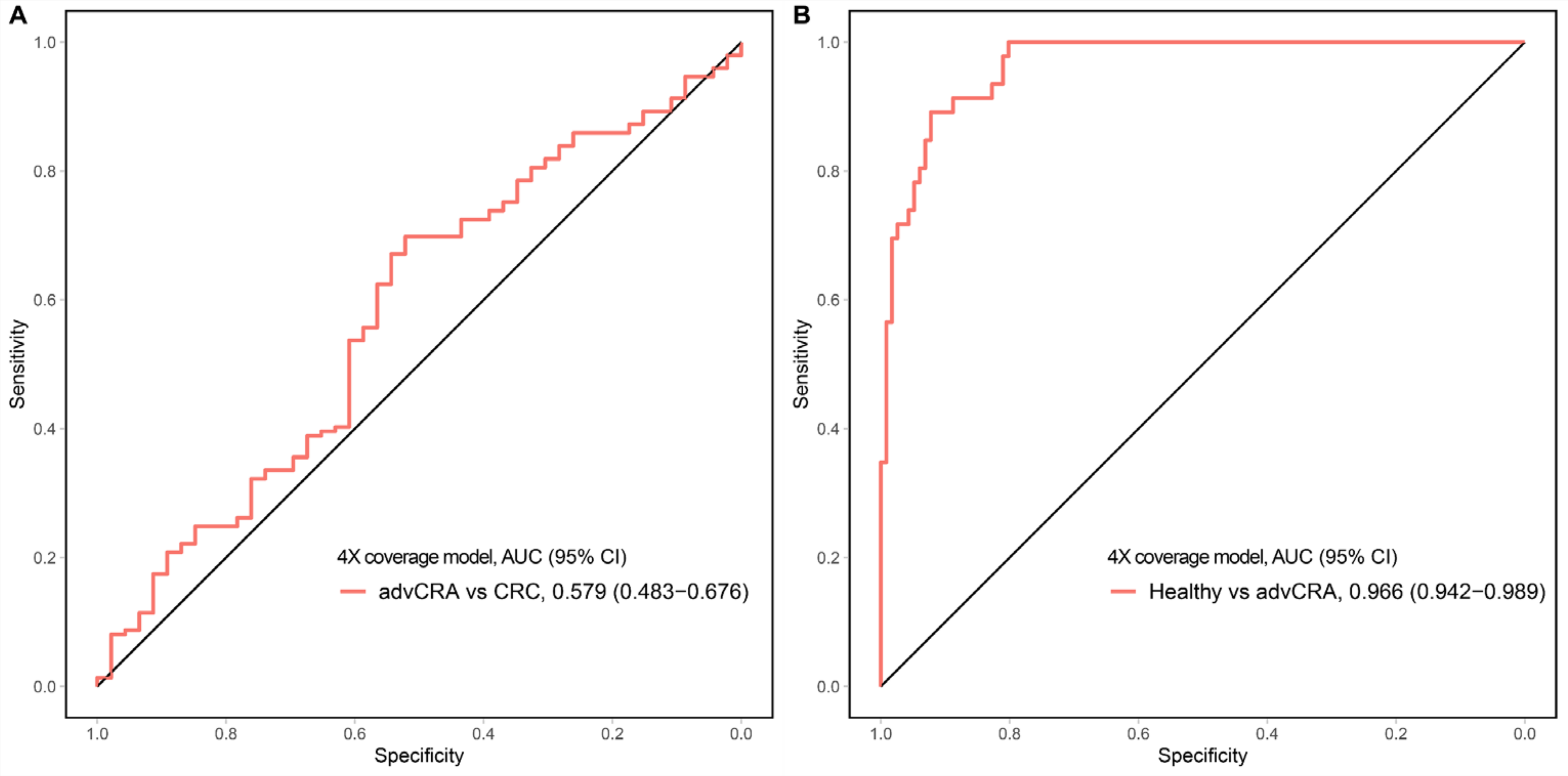

Figure S2. Evaluation of models distinguishing advCRA from early-stage CRC or healthy controls.

A ROC curve showing a separate model, which was constructed using 4x coverage WGS data, in distinguishing advCRA from early-stage CRC patients in the test cohort. **B** ROC curve showing the overall performance of a separate model, which was constructed using 4x coverage WGS data, in distinguishing advCRA from healthy controls in the test cohort.

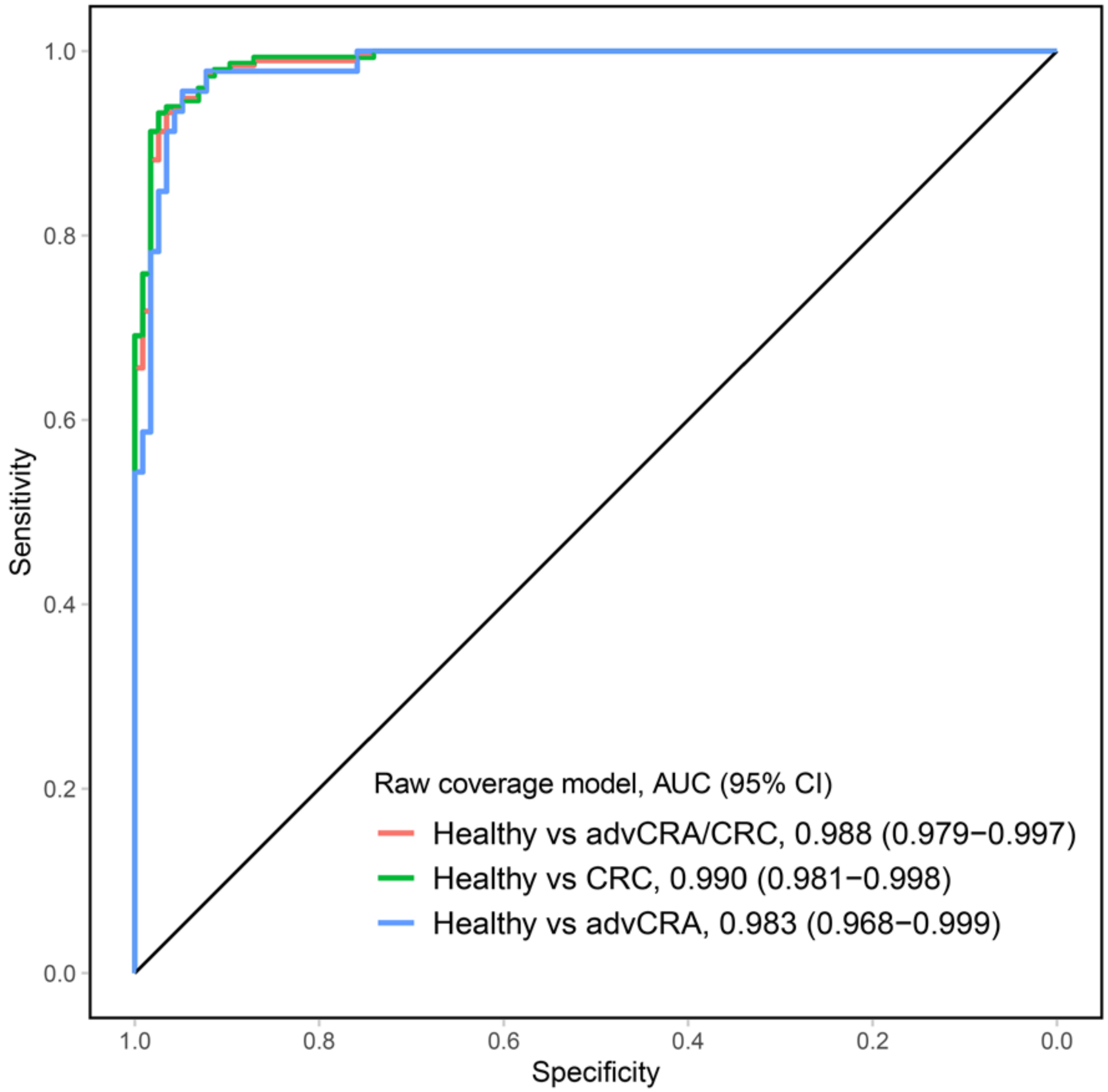

Figure S3. Evaluation of model constructed using raw coverage WGS data.

ROC curves showing the overall performance of the predictive model, which was constructed using raw coverage WGS data, in distinguishing advCRA/early-stage CRC patients from healthy controls in the test cohort.

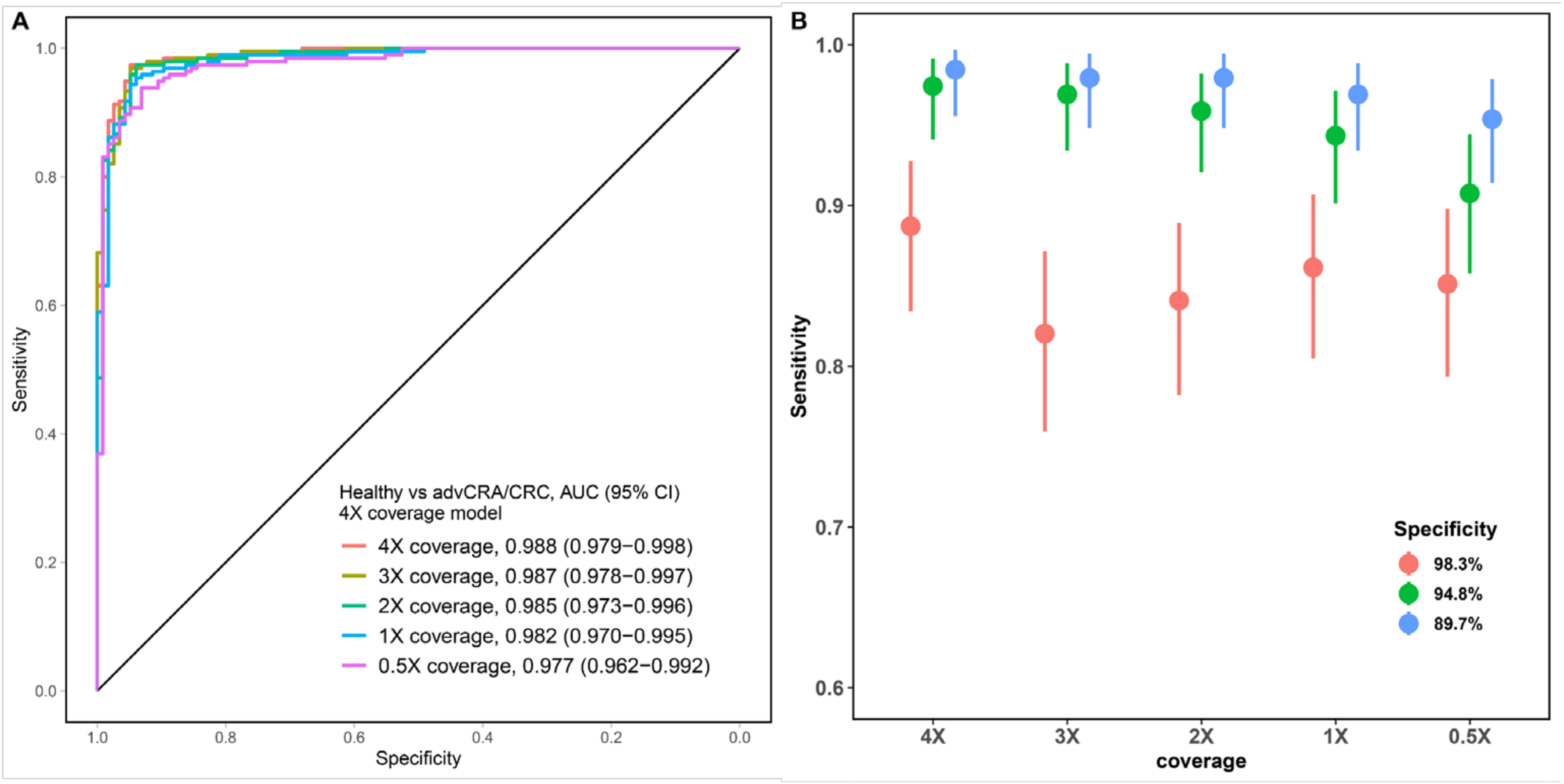

Figure S4. Evaluation of a multi-dimensional model detecting advCRA/early-stage CRC.

A ROC curves of a limit of detection analysis, the 4X coverage model was evaluated using WGS data downsampled to 3X, 2X, 1X and 0.5X. **B** Dot plot of 4X coverage model sensitivity in detecting advCRA/early-stage CRC using 4X, 3X, 2X, 1X and 0.5X WGS data, at 98.3%, 94.8% and 89.7% specificity for healthy controls. The error bars represented 95% confidence interval.

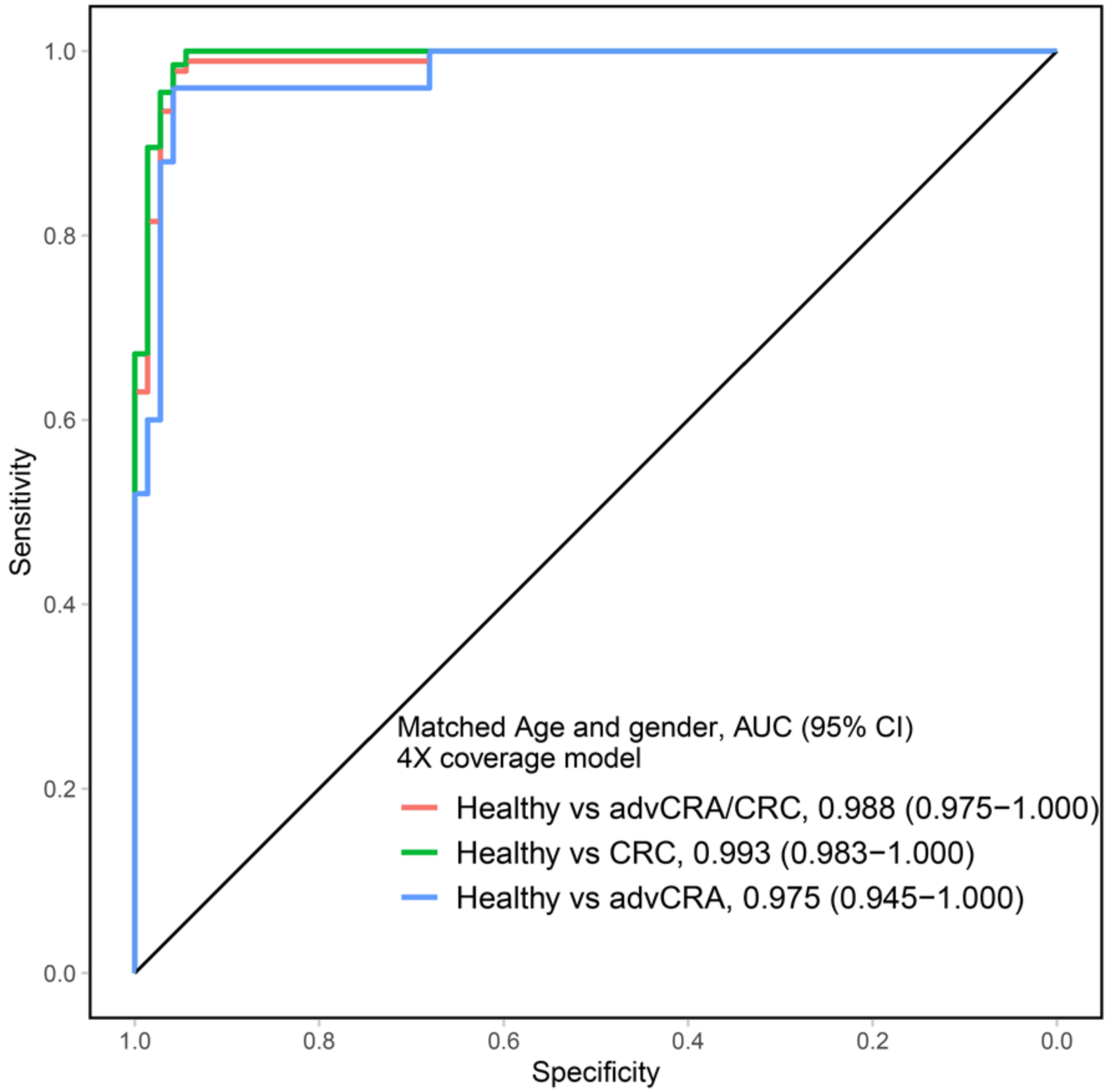

Figure S5. Evaluation of age and gender matched groups in the test cohort.

ROC curves showing model performances in distinguishing advCRA/early-stage CRC from a subset consisting of 25 advCRA, 67 early-stage CRC and 72 healthy controls, which were selected from the test cohort with matching age and gender.

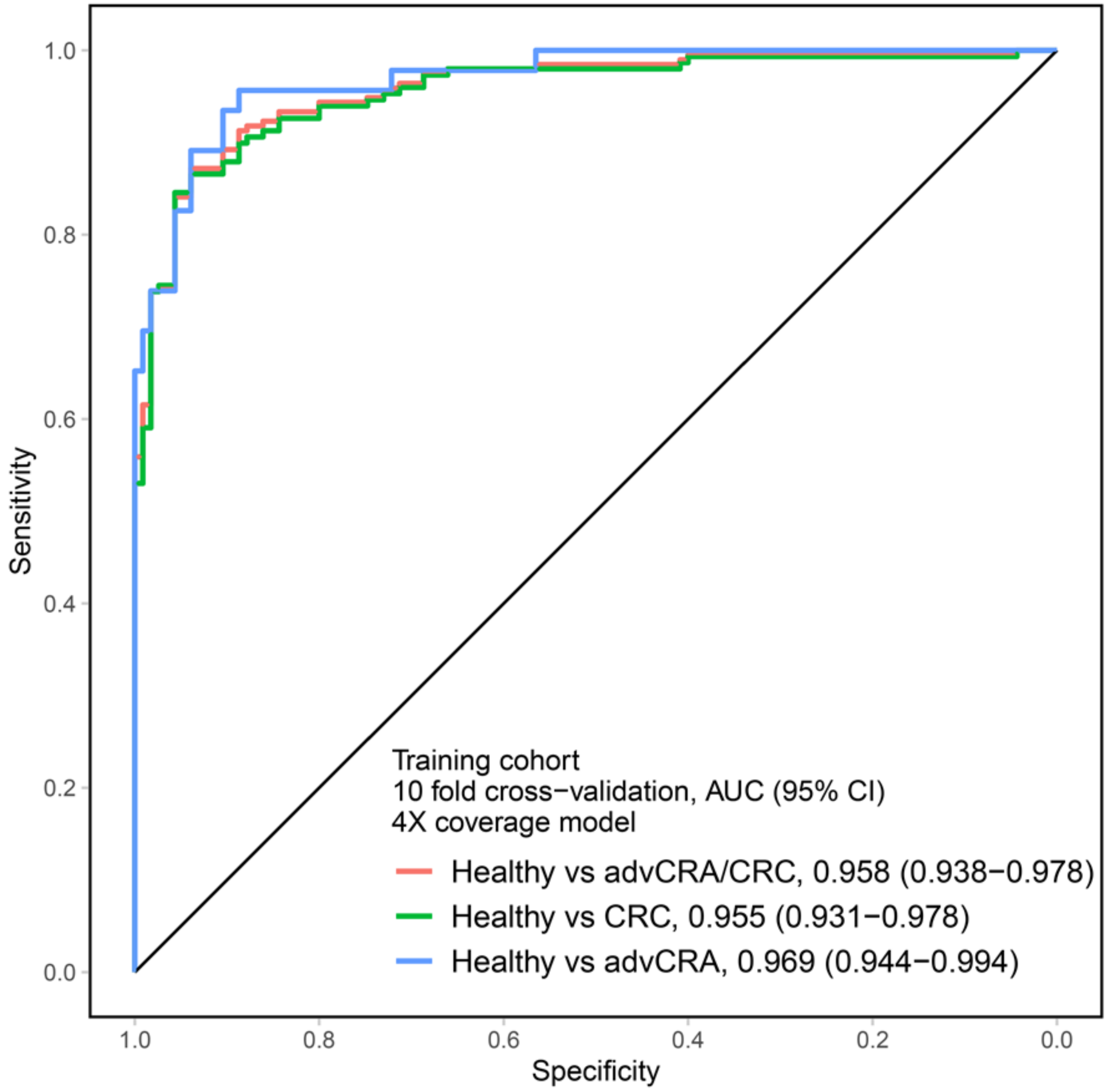

Figure S6. Evaluation of model using 10-fold cross-validation score of the training cohort.

ROC curves showing the overall performance of the predictive model, using 10-fold cross-validation score of the training cohort, in distinguishing advCRA/early-stage CRC patients from healthy controls.

Table S1. Performances evaluation of base models using different features.

Healthy vs advCRA/CRC	Test dataset	
	Sensitivity	Specificity
**Stacked model**	97.4% (94.1-99.2%)	94.8% (89.1-98.1%)
**FSR**	57.4% (50.2-64.5%)	94.8% (89.1-98.1%)
**FSD**	55.9% (48.6-63%)	94.8% (89.1-98.1%)
**EDM**	89.2% (84-93.2%)	94.8% (89.1-98.1%)
**BPM**	86.2% (80.5-90.7%)	94.8% (89.1-98.1%)
**CNV**	72.3% (65.5-78.5%)	94.8% (89.1-98.1%)
**Orig. Frag**	42.1% (35-49.3%)	94.8% (89.1-98.1%)
**Orig. Motif**	86.7% (81.1-91.1%)	94.8% (89.1-98.1%)

Table S2. Evaluating performances of model constructed by raw depth data in the test dataset

Healthy vs advCRA/CRC			
Test dataset		Actual	
		advCRA/CRC	Healthy
Predict	advCRA/CRC	185	6
	Healthy	10	110
Sensitivity (95% CI)		94.9% (90.8-97.5%)	
Specificity (95% CI)		94.8% (89.1-98.1%)	
PPV (95% CI)		96.9% (93.3-98.8%)	
NPV (95% CI)		91.7% (85.2-95.9%)	
Accuracy (95% CI)		94.9% (91.8-97%)	
**Healthy vs advCRA**			
Test dataset		Actual	
		advCRA	Healthy
Predict	advCRA	44	6
	Healthy	2	110
Sensitivity (95% CI)		95.7% (85.2-99.5%)	
Specificity (95% CI)		94.8% (89.1-98.1%)	
PPV (95% CI)		88% (75.7-95.5%)	
NPV (95% CI)		98.2% (93.7-99.8%)	
Accuracy (95% CI)		95.1% (90.5-97.8%)	
**Healthy vs CRC**			
Test dataset		Actual	
		CRC	Healthy
Predict	CRC	141	6
	Healthy	8	110
Sensitivity (95% CI)		94.6% (89.7-97.7%)	
Specificity (95% CI)		94.8% (89.1-98.1%)	
PPV (95% CI)		95.9% (91.3-98.5%)	
NPV (95% CI)		93.2% (87.1-97%)	
Accuracy (95% CI)		94.7% (91.3-97.1%)	

Table S3. Participant demographics and baseline characteristics.

	Training (N=310)			Test (N=311)		
	Healthy	advCRA	CRC	Healthy	advCRA	CRC
**Total, n (%)**	115 (37.1%)	46 (14.8%)	149 (48.1%)	116 (37.3%)	46 (14.8%)	149 (47.9%)
**Age, Mean ± SD**	45.5 ± 14.3	58.7 ± 11.3	61.1 ± 9.5	46.4 ± 13.4	58.0 ± 11.0	62.1 ± 9.8
**Sex, n (%)**						
Female, n (%)	70 (60.9%)	16 (34.8%)	61 (40.9%)	66 (56.9%)	26 (56.5%)	61 (40.9%)
**Smoke history, n (%)**	-	1 (2.2%)	16 (10.7%)	-	2 (4.4%)	5 (3.4%)
**Drink history, n (%)**	-	3 (6.5%)	13 (8.7%)	-	2 (4.4%)	6 (4.0%)
**Diabetes, n (%)**	-	2 (4.4%)	17 (11.4%)	-	5 (10.9%)	15 (10.1%)
**High blood pressure, n (%)**	-	12 (26.1%)	35 (23.5%)	-	11 (23.9%)	48 (32.2%)
**Stage TNM, n (%)**						
0	-	-	17 (11.4%)	-	-	17 (11.4%)
I	-	-	132 (88.6%)	-	-	132 (88.6%)
**FOBT, n (%)**						
Pos	-	11 (23.9%)	55 (36.9%)	-	11 (23.9%)	51 (34.2%)
Neg	-	20 (43.5%)	52 (34.9%)	-	21 (45.7%)	43 (28.9%)
NA	-	15 (32.6%)	42 (28.2%)	-	14 (30.4%)	55 (36.9%)
**CEA, n (%)**						
Pos	-	5 (10.9%)	16 (10.7%)	-	3 (6.5%)	24 (16.1%)
Neg	-	40 (87.0%)	133 (89.3%)	-	43 (93.5%)	125 (83.9%)
NA	-	1 (2.1%)	-	-	-	-
**CA199, n (%)**						
Pos	-	2 (4.4%)	12 (8.1%)	-	5 (10.9%)	13 (8.7%)
Neg	-	43 (93.5%)	137 (92.0%)	-	41 (89.1%)	136 (91.3%)
NA	-	1 (2.1%)	-	-	-	-
**Location, n (%)**						
Left	-	14 (30.4%)	36 (24.2%)	-	8 (17.3%)	38 (25.5%)
Colon	-	18 (39.2%)	85 (57.0%)	-	21 (45.7%)	85 (57.0%)
Right	-	14 (30.4%)	28 (18.8%)	-	17 (37.0%)	26 (17.4%)
**Size (cm), n (%)**						
< 3	-	25 (54.3%)	81 (54.4%)	-	18 (39.1%)	75 (50.3%)
>= 3	-	21 (45.7%)	68 (45.6%)	-	28 (60.9%)	74 (49.7%)
**Grade, n (%)**						
High	-	24 (52.2%)	53 (35.6%)	-	23 (50.0%)	44 (29.5%)
Intermediate	-	-	77 (51.7%)	-	-	90 (60.4%)
Low	-	21 (45.7%)	19 (12.8%)	-	22 (47.8%)	15 (10.1%)
**Endoscopic, n (%)**						
pedunculated	-	15 (32.6%)	-	-	14 (30.4%)	-
sessile	-	31 (67.4%)	-	-	32 (69.6%)	-
**Vascular invasion, n (%)**						
Pos	-	-	14 (9.4%)	-	-	10 (6.7%)
Neg	-	-	118 (79.2%)	-	-	122 (81.9%)
NA	-	-	17 (11.4%)	-	-	17 (11.4%)
**Perineural invasion, n (%)**						
Pos	-	-	10 (6.1%)	-	-	3 (2.0%)
Neg	-	-	122 (81.9%)	-	-	129 (86.6%)

NA	-	-	17 (11.4%)	-	-	17 (11.4%)
**MMR, n (%)**						
dMMR	-	-	8 (5.4%)	-	-	9 (6.0%)
pMMR	-	-	108 (72.5%)	-	-	113 (75.8%)
NA	-	-	33 (22.1%)	-	-	27 (18.2%)
**KRAS, n (%)**						
Mut	-	-	41 (27.5%)	-	-	51 (34.2%)
WT	-	-	70 (47.0%)	-	-	68 (45.6%)
NA	-	-	38 (25.5%)	-	-	30 (20.2%)
**NRAS, n (%)**						
Mut	-	-	4 (2.7%)	-	-	6 (4.0%)
WT	-	-	107 (71.8%)	-	-	113 (75.8%)
NA	-	-	38 (25.5%)	-	-	30 (20.2%)
**BRAF, n (%)**						
Mut	-	-	4 (2.7%)	-	-	6 (4.0%)
WT	-	-	107 (71.8%)	-	-	113 (75.8%)
NA	-	-	38 (25.5%)	-	-	30 (20.2%)

Table S4. Clinical information of the colorectal advanced adenoma (advCRA) and Adenocarcinoma (CRC) patients

Cohort	PatientID	Type	Sex	Age	Smoke history	Drinking history	Height (cm)	Weight (kg)	BMI	Diabetes	High blood pressure	Location	Size (cm)	AJCC	T	M	N	FOBT	CA199	CEA	Endoscopic	Grade	KRAS	NRAS	BRAF	MMR
Training	Training_001	advCRA	F	36	No	No	165	51	18.7328	No	No	Colon	3	NA	NA	NA	NA	NA	Neg	Neg	sessile	Low	NA	NA	NA	NA
Training	Training_002	advCRA	F	44	No	No	152	56.5	24.4546	No	No	Right	2.2	NA	NA	NA	NA	NA	Neg	Neg	sessile	High	NA	NA	NA	NA
Training	Training_003	advCRA	F	52	No	No	164	74	28.9063	No	No	Right	2.5	NA	NA	NA	NA	NA	Neg	Neg	sessile	High	NA	NA	NA	NA
Training	Training_004	advCRA	M	60	No	No	171	80	27.3588	No	Yes	Right	1.1	NA	NA	NA	NA	Neg	Neg	Neg	pedunculated	Low	NA	NA	NA	NA
Training	Training_005	advCRA	M	65	No	No	170	80	27.6817	Yes	Yes	Right	1.3	NA	NA	NA	NA	Neg	Neg	Neg	sessile	Low	NA	NA	NA	NA
Training	Training_006	advCRA	M	50	No	No	168	75	26.5731	No	No	Right	2.8	NA	NA	NA	NA	Neg	Neg	Neg	pedunculated	High	NA	NA	NA	NA
Training	Training_007	advCRA	M	70	No	No	169	71	24.8591	No	No	Left	2.8	NA	NA	NA	NA	Neg	Neg	Neg	sessile	High	NA	NA	NA	NA
Training	Training_008	advCRA	F	40	No	No	160	70	27.3438	No	No	Right	4	NA	NA	NA	NA	Pos	Neg	Neg	pedunculated	Low	NA	NA	NA	NA
Training	Training_009	advCRA	M	72	Yes	Yes	173	55	16.7062	No	No	Colon	1.2	NA	NA	NA	NA	Neg	Neg	Neg	sessile	High	NA	NA	NA	NA
Training	Training_010	advCRA	M	69	No	No	173	82.5	27.5652	No	Yes	Right	4	NA	NA	NA	NA	NA	Neg	Neg	sessile	Low	NA	NA	NA	NA
Training	Training_011	advCRA	F	62	No	No	162	50.5	19.2425	No	Yes	Colon	2	NA	NA	NA	NA	Neg	Neg	Neg	sessile	Low	NA	NA	NA	NA
Training	Training_012	advCRA	M	61	No	No	165	62.5	22.9568	No	No	Left	2	NA	NA	NA	NA	NA	Pos	Neg	pedunculated	Low	NA	NA	NA	NA
Training	Training_013	advCRA	M	54	No	Yes	168	70	24.8016	No	Yes	Colon	2.5	NA	NA	NA	NA	Neg	Neg	Pos	pedunculated	High	NA	NA	NA	NA
Training	Training_014	advCRA	F	67	No	No	157	63	25.5588	No	No	Colon	3	NA	NA	NA	NA	NA	Neg	Neg	sessile	Low	NA	NA	NA	NA
Training	Training_015	advCRA	F	69	No	No	165	55	20.202	No	No	Left	2.2	NA	NA	NA	NA	Neg	Neg	Neg	sessile	Low	NA	NA	NA	NA
Training	Training_016	advCRA	F	65	No	No	175	65	21.2245	No	No	Colon	2.5	NA	NA	NA	NA	Pos	Neg	Neg	pedunculated	High	NA	NA	NA	NA
Training	Training_017	advCRA	M	59	No	No	173	85	28.4005	No	Yes	Colon	4	NA	NA	NA	NA	Pos	Neg	Neg	sessile	High	NA	NA	NA	NA
Training	Training_018	advCRA	F	38	No	No	168	54	19.1327	No	No	Left	2.5	NA	NA	NA	NA	NA	Neg	Neg	pedunculated	Low	NA	NA	NA	NA
Training	Training_019	advCRA	M	61	No	No	164	60	22.3081	No	No	Left	2	NA	NA	NA	NA	NA	Neg	Neg	sessile	Low	NA	NA	NA	NA
Training	Training_020	advCRA	F	43	No	No	160	60.5	23.6328	No	No	Left	5	NA	NA	NA	NA	NA	Neg	Neg	sessile	High	NA	NA	NA	NA
Training	Training_021	advCRA	M	63	No	Yes	176	80	25.8264	No	No	Right	3.5	NA	NA	NA	NA	NA	Neg	Neg	sessile	High	NA	NA	NA	NA
Training	Training_022	advCRA	M	48	No	No	165	63	23.1405	No	No	Colon	1.5	NA	NA	NA	NA	NA	Neg	Neg	pedunculated	Low	NA	NA	NA	NA
Training	Training_023	advCRA	F	72	No	No	162	60	22.8624	No	No	Right	6	NA	NA	NA	NA	Neg	Neg	Neg	sessile	Low	NA	NA	NA	NA
Training	Training_024	advCRA	M	59	No	No	170	62	21.4533	No	No	Left	2.5	NA	NA	NA	NA	Pos	Neg	Neg	sessile	High	NA	NA	NA	NA
Training	Training_025	advCRA	F	56	No	No	162	49	18.6709	No	No	Colon	4	NA	NA	NA	NA	NA	Neg	Neg	sessile	NA	NA	NA	NA	NA
Training	Training_026	advCRA	M	74	No	No	170	70	24.2215	No	No	Colon	2	NA	NA	NA	NA	Neg	Neg	Neg	pedunculated	High	NA	NA	NA	NA
Training	Training_027	advCRA	M	69	No	No	170	73	25.2595	No	Yes	Left	2.7	NA	NA	NA	NA	Neg	Neg	Pos	sessile	High	NA	NA	NA	NA
Training	Training_028	advCRA	M	66	No	No	180	55	16.9753	No	No	Colon	6	NA	NA	NA	NA	Neg	Neg	Neg	pedunculated	High	NA	NA	NA	NA
Training	Training_029	advCRA	M	56	No	No	178	62	19.5682	No	Yes	Left	3	NA	NA	NA	NA	Pos	Neg	Neg	pedunculated	Low	NA	NA	NA	NA
Training	Training_030	advCRA	M	65	No	No	165	72	26.4463	No	No	Right	3.3	NA	NA	NA	NA	Neg	Neg	Pos	sessile	High	NA	NA	NA	NA
Training	Training_031	advCRA	M	64	No	No	170	60	20.7612	Yes	No	Colon	2	NA	NA	NA	NA	Neg	Neg	Neg	sessile	High	NA	NA	NA	NA
Training	Training_032	advCRA	M	64	No	No	177	68	21.7051	No	Yes	Left	4	NA	NA	NA	NA	NA	Neg	Neg	pedunculated	Low	NA	NA	NA	NA
Training	Training_033	advCRA	M	48	No	No	178	80	24.6914	No	No	Left	5	NA	NA	NA	NA	Pos	Neg	Pos	sessile	High	NA	NA	NA	NA
Training	Training_034	advCRA	M	72	No	No	166	71	25.7657	No	No	Right	2.5	NA	NA	NA	NA	Pos	NA	NA	sessile	High	NA	NA	NA	NA
Training	Training_035	advCRA	F	55	No	No	158	60	24.0346	No	No	Colon	1.8	NA	NA	NA	NA	Neg	Neg	Neg	sessile	High	NA	NA	NA	NA
Training	Training_036	advCRA	M	62	No	No	183	81	24.187	No	No	Colon	3.2	NA	NA	NA	NA	Neg	Neg	Neg	sessile	High	NA	NA	NA	NA
Training	Training_037	advCRA	M	64	No	No	160	70	27.3438	No	Yes	Colon	1.8	NA	NA	NA	NA	Neg	Neg	Neg	pedunculated	Low	NA	NA	NA	NA
Training	Training_038	advCRA	M	51	No	No	165	64	23.5078	No	No	Left	4	NA	NA	NA	NA	NA	Neg	Neg	pedunculated	High	NA	NA	NA	NA
Training	Training_039	advCRA	M	63	No	No	170	80	27.6817	No	No	Right	3.5	NA	NA	NA	NA	Neg	Pos	Neg	sessile	Low	NA	NA	NA	NA
Training	Training_040	advCRA	F	72	No	No	158	44	17.6254	No	Yes	Colon	1.5	NA	NA	NA	NA	Neg	Neg	Pos	sessile	Low	NA	NA	NA	NA
Training	Training_041	advCRA	M	54	No	No	170	67	23.1834	No	No	Colon	3.5	NA	NA	NA	NA	NA	Neg	Neg	sessile	High	NA	NA	NA	NA
Training	Training_042	advCRA	F	36	No	No	160	52.5	20.5078	No	No	Right	5	NA	NA	NA	NA	Pos	Neg	Neg	pedunculated	Low	NA	NA	NA	NA
Training	Training_043	advCRA	F	75	No	No	155	58	24.1415	No	Yes	Right	2.8	NA	NA	NA	NA	Pos	Neg	Neg	sessile	Low	NA	NA	NA	NA
Training	Training_044	advCRA	M	65	No	No	173	80	26.7299	No	No	Colon	5	NA	NA	NA	NA	Pos	Neg	Neg	sessile	High	NA	NA	NA	NA
Training	Training_045	advCRA	M	29	No	No	172	75	25.3515	No	No	Left	3.3	NA	NA	NA	NA	Pos	Neg	Neg	sessile	High	NA	NA	NA	NA
Training	Training_046	advCRA	M	63	No	No	178	80	25.2493	No	No	Left	1.8	NA	NA	NA	NA	Neg	Neg	Neg	sessile	Low	NA	NA	NA	NA
Training	Training_047	CRC	F	65	No	No	170	59	20.4152	No	No	Right	2.5	I	1	0	0	Pos	Pos	Pos	NA	High	WT	WT	WT	pMMR
Training	Training_048	CRC	M	61	No	No	178	72	22.7244	No	No	Colon	2	I	1	0	0	NA	Neg	Neg	NA	High	Mut	WT	WT	pMMR
Training	Training_049	CRC	M	75	No	No	166	68	24.677	Yes	Yes	Right	5	I	1	0	0	Pos	Neg	Neg	NA	High	NA	NA	NA	NA
Training	Training_050	CRC	F	66	No	No	147	40	18.5108	No	No	Colon	2.5	I	2	0	0	Pos	Neg	Neg	NA	Low	Mut	WT	WT	pMMR
Training	Training_051	CRC	F	68	No	No	163	65	24.4646	Yes	No	Left	3	I	2	0	0	NA	Neg	Neg	NA	Intermediate	WT	Mut	WT	pMMR
Training	Training_052	CRC	M	70	No	No	168	60	21.2585	No	Yes	Colon	1.5	I	2	0	0	Pos	Neg	Neg	NA	Low	WT	WT	WT	pMMR
Training	Training_053	CRC	F	57	No	No	157	56	22.719	No	No	Colon	2.5	I	2	0	0	NA	Neg	Neg	NA	Intermediate	Mut	WT	WT	pMMR
Training	Training_054	CRC	F	59	No	No	155	60	24.974	No	No	Colon	2.5	I	1	0	0	NA	Neg	Neg	NA	Intermediate	WT	WT	WT	pMMR
Training	Training_055	CRC	M	72	Yes	Yes	169	60	21.0077	Yes	Yes	Colon	2.5	I	2	0	0	Pos	Neg	Neg	NA	Intermediate	Mut	WT	WT	pMMR
Training	Training_056	CRC	M	54	No	No	169	66.5	23.2835	No	No	Colon	1	I	1	0	0	NA	Neg	Neg	NA	Intermediate	WT	WT	WT	pMMR
Training	Training_057	CRC	F	68	No	No	157	75	30.4272	No	No	Colon	3.5	I	1	0	0	Pos	Neg	Neg	NA	High	NA	NA	NA	NA
Training	Training_058	CRC	M	56	Yes	No	165	76	27.9155	No	Yes	Colon	2	I	2	0	0	Neg	Neg	Neg	NA	Intermediate	WT	WT	WT	pMMR
Training	Training_059	CRC	M	68	No	No	180	87	26.8519	Yes	Yes	Left	3	I	2	0	0	NA	Pos	Pos	NA	Intermediate	WT	WT	WT	pMMR
Training	Training_060	CRC	M	39	No	No	165	60	22.0386	No	No	Colon	1.6	I	2	0	0	Pos	Neg	Neg	NA	Intermediate	NA	NA	NA	NA
Training	Training_061	CRC	F	41	No	No	150	50.5	22.4444	No	No	Left	0.8	I	1	0	0	Neg	Neg	Neg	NA	Intermediate	NA	NA	NA	NA
Training	Training_062	CRC	M	50	No	No	174	60	19.8177	No	No	Right	4.5	I	1	0	0	NA	Pos	Neg	NA	High	NA	NA	NA	NA
Training	Training_063	CRC	F	50	No	No	165	78	28.6501	No	No	Left	3.5	I	2	0	0	Neg	Neg	Pos	NA	Intermediate	WT	WT	WT	pMMR
Training	Training_064	CRC	M	69	No	No	170	80	27.6817	No	Yes	Right	2.5	I	2	0	0	Pos	Neg	Neg	NA	Intermediate	WT	WT	WT	dMMR
Training	Training_065	CRC	F	59	No	No	160	65	25.3906	No	No	Colon	4.5	I	2	0	0	Pos	Neg	Neg	NA	Intermediate	Mut	WT	WT	pMMR
Training	Training_066	CRC	F	49	No	No	156	59	24.2439	No	No	Colon	2.5	I	2	0	0	Pos	Neg	Neg	NA	Intermediate	WT	WT	WT	pMMR
Training	Training_067	CRC	M	61	No	No	170	67.5	23.3564	No	No	Right	3	I	2	0	0	Neg	Neg	Neg	NA	High	Mut	WT	WT	pMMR
Training	Training_068	CRC	M	58	No	No	173	89	29.737	Yes	Yes	Left	1.7	I	2	0	0	Pos	Neg	Neg	NA	Intermediate	WT	WT	WT	pMMR
Training	Training_069	CRC	M	57	No	No	175	79	25.7959	No	No	Colon	4.5	I	1	0	0	Neg	Neg	Neg	NA	Intermediate	Mut	WT	WT	pMMR
Training	Training_070	CRC	F	70	No	No	162	54	20.5761	No	No	Colon	3	I	2	0	0	Pos	Neg	Neg	NA	Intermediate	Mut	WT	WT	pMMR
Training	Training_071	CRC	M	63	Yes	Yes	170	72	24.9135	No	No	Left	2.5	I	2	0	0	Pos	Neg	Pos	NA	Low	WT	WT	WT	pMMR
Training	Training_072	CRC	F	63	No	No	163	54	20.3244	No	No	Colon	1.8	I	2	0	0	Neg	Neg	Neg	NA	Intermediate	Mut	WT	WT	pMMR
Training	Training_073	CRC	M	74	No	No	155	50	20.8117	Yes	No	Right	2.4	I	1	0	0	Pos	Pos	Pos	NA	Intermediate	Mut	WT	WT	pMMR
Training	Training_074	CRC	M	62	No	No	160	60	23.4375	No	Yes	Right	1	I	1	0	0	Pos	Neg	Neg	NA	Intermediate	WT	WT	WT	pMMR
Training	Training_075	CRC	F	62	No	No	155	55	22.8928	No	No	Colon	6	I	2	0	0	Pos	Neg	Neg	NA	Intermediate	WT	WT	WT	pMMR
Training	Training_076	CRC	M	56	No	No	165	60	22.0386	No	No	Colon	1	I	1	0	0	Neg	Neg	Neg	NA	High	NA	NA	NA	NA
Training	Training_077	CRC	M	67	No	No	169	66	23.1084	No	Yes	Left	1.8	I	1	0	0	Pos	Neg	Neg	NA	High	WT	WT	WT	pMMR
Training	Training_078	CRC	M	55	No	No	175	75	24.4898	No	No	Right	7	I	2	0	0	Neg	Neg	Neg	NA	Intermediate	WT	WT	WT	dMMR
Training	Training_079	CRC	M	70	No	No	160	65	25.3906	No	No	Colon	3.5	I	2	0	0	Pos	Neg	Pos	NA	Intermediate	Mut	WT	WT	pMMR
Training	Training_080	CRC	F	56	No	No	165	52	19.1001	No	No	Colon	4	I	1	0	0	Pos	Neg	Neg	NA	Intermediate	Mut	WT	WT	pMMR
Training	Training_081	CRC	F	51	No	No	160	54.8	21.4063	No	No	Colon	2	I	1	0	0	Pos	Neg	Neg	NA	Intermediate	NA	NA	NA	pMMR
Training	Training_082	CRC	M	67	No	No	168	65	23.03	No	Yes	Right	2.5	I	1	0	0	Pos	Neg	Neg	NA	Low	Mut	WT	WT	pMMR
Training	Training_083	CRC	F	57	No	No	145	59	28.0618	No	No	Colon	2.5	I	2	0	0	Pos	Neg	Neg	NA	Low	Mut	WT	WT	pMMR
Training	Training_084	CRC	F	53	No	No	160	71	27.7344	No	No	Colon	3.5	I	1	0	0	NA	Neg	Neg	NA	Intermediate	Mut	WT	WT	pMMR
Training	Training_085	CRC	M	74	No	No	169	75	26.2596	No	No	Left	2.3	I	2	0	0	NA	Neg	Neg	NA	Low	WT	WT	WT	pMMR
Training	Training_086	CRC	F	55	No	No	163	58	21.83	Yes	Yes	Colon	2	I	2	0	0	Neg	Neg	Neg	NA	Intermediate	Mut	WT	WT	pMMR
Training	Training_087	CRC	M	62	No	No	165	60	22.0386	No	No	Colon	2.5	I	1	0	0	Pos	Neg	Neg	NA	Intermediate	NA	NA	NA	NA
Training	Training_088	CRC	M	64	Yes	Yes	172	70	23.6614	No	No	Colon	4	I	2	0	0	Pos	Neg	Neg	NA	Low	WT	WT	WT	pMMR
Training	Training_089	CRC	F	53	No	No	155	56	23.3091	No	No	Left	3.5	I	2	0	0	NA	Neg	Neg	NA	Intermediate	Mut	WT	WT	dMMR
Training	Training_090	CRC	F	62	No	No	167	79	28.3266	No	No	Right	1.5	I	1	0	0	Neg	Neg	Neg	NA	High	WT	WT	WT	pMMR
Training	Training_091	CRC	M	75	No	No	172	60	20.2812	No	No	Colon	3	I	2	0	0	Pos	Pos	Pos	NA	Intermediate	WT	WT	WT	pMMR
Training	Training_092	CRC	M	68	No	No	170	70	24.2215	No	No	Colon	5	I	2	0	0	NA	Neg	Neg	NA	Intermediate	WT	WT	WT	pMMR
Training	Training_093	CRC	F	64	No	No	155	64	26.6389	No	No	Right	2	I	1	0	0	Neg	Neg	Neg	NA	Intermediate	Mut	WT	WT	pMMR
Training	Training_094	CRC	F	42	No	No	158	60.9	24.3951	No	No	Colon	3.5	I	1	0	0	Pos	Neg	Neg	NA	Intermediate	Mut	WT	WT	dMMR
Training	Training_095	CRC	F	70	No	No	153	54	23.0681	No	No	Colon	2.8	I	2	0	0	Pos	Neg	Neg	NA	Intermediate	Mut	WT	WT	pMMR
Training	Training_096	CRC	M	71	No	No	176	70	22.5981	No	No	Colon	2.5	I	2	0	0	Neg	Neg	Neg	NA	Low	WT	WT	WT	pMMR
Training	Training_097	CRC	F	77	No	No	150	46.2	20.5333	Yes	Yes	Colon	3.5	I	1	0	0	NA	Neg	Neg	NA	High	WT	WT	WT	pMMR
Training	Training_098	CRC	M	39	No	No	178	83	26.1962	No	No	Left	2.5	I	2	0	0	Pos	Neg	Neg	NA	Low	Mut	WT	WT	pMMR
Training	Training_099	CRC	M	64	No	No	165	65	23.8751	No	No	Colon	1	I	1	0	0	Neg	Neg	Pos	NA	Intermediate	WT	WT	WT	pMMR
Training	Training_100	CRC	M	72	No	No	170	66	22.8374	No	Yes	Right	5	I	2	0	0	NA	Pos	Neg	NA	High	Mut	WT	WT	pMMR
Training	Training_101	CRC	M	59	No	No	172	60	20.2812	No	Yes	Colon	4	I	2	0	0	NA	Neg	Neg	NA	High	WT	WT	WT	pMMR
Training	Training_102	CRC	M	59	No	No	169	81	28.3604	No	No	Left	2	I	1	0	0	Neg	Neg	Pos	NA	High	Mut	WT	WT	pMMR
Training	Training_103	CRC	F	58	No	No	154	50	21.0828	No	No	Left	2	I	1	0	0	Pos	Neg	Neg	NA	Intermediate	WT	WT	WT	pMMR
Training	Training_104	CRC	M	55	No	No	170	67	23.1834	No	No	Colon	1	I	1	0	0	Neg	Neg	Neg	NA	Intermediate	NA	NA	NA	NA
Training	Training_105	CRC	M	77	No	No	175	76	24.8163	No	No	Left	5	I	2	0	0	Pos	Neg	Neg	NA	Intermediate	WT	WT	Mut	pMMR
Training	Training_106	CRC	F	58	No	No	162	63	24.0055	No	Yes	Right	3	I	2	0	0	Neg	Neg	Neg	NA	Intermediate	Mut	WT	WT	pMMR
Training	Training_107	CRC	F	67	No	No	155	55	22.8928	No	Yes	Colon	3.1	I	2	0	0	Pos	Neg	Neg	NA	Intermediate	NA	NA	NA	pMMR
Training	Training_108	CRC	F	62	No	No	160	64	25	No	No	Left	1.5	I	2	0	0	NA	Neg	Neg	NA	Low	WT	WT	WT	pMMR
Training	Training_109	CRC	M	54	Yes	Yes	160	66	25.7813	No	Yes	Colon	2	I	2	0	0	Neg	Neg	Neg	NA	Intermediate	Mut	WT	WT	pMMR
Training	Training_110	CRC	M	47	Yes	No	172	71	23.9995	No	No	Colon	3	I	2	0	0	Pos	Pos	Pos	NA	Intermediate	Mut	WT	WT	pMMR
Training	Training_111	CRC	M	63	No	No	180	67.5	20.8333	No	No	Left	4	I	2	0	0	Neg	Neg	Pos	NA	Intermediate	WT	WT	WT	pMMR
Training	Training_112	CRC	M	41	No	No	166	74	26.8544	No	No	Colon	4	I	2	0	0	NA	Neg	Neg	NA	High	Mut	WT	WT	pMMR
Training	Training_113	CRC	F	65	No	No	158	60	24.0346	No	No	Colon	3	I	2	0	0	Neg	Neg	Neg	NA	Intermediate	WT	WT	WT	pMMR
Training	Training_114	CRC	M	53	Yes	Yes	170	77	26.6436	No	No	Left	3	I	2	0	0	Neg	Neg	Neg	NA	Low	WT	WT	WT	pMMR
Training	Training_115	CRC	F	60	No	No	158	60	24.0346	No	No	Right	5.6	I	2	0	0	Pos	Neg	Neg	NA	Low	Mut	WT	WT	pMMR
Training	Training_116	CRC	F	61	No	No	157	57.5	23.3275	No	No	Colon	1.8	I	2	0	0	Neg	Neg	Neg	NA	High	Mut	WT	WT	pMMR
Training	Training_117	CRC	F	43	No	No	160	45	17.5781	No	No	Colon	3.2	I	2	0	0	Neg	Neg	Neg	NA	Intermediate	WT	WT	WT	pMMR
Training	Training_118	CRC	M	54	No	No	165	69	25.3444	No	No	Colon	4.5	I	2	0	0	Neg	Neg	Neg	NA	Intermediate	WT	WT	WT	pMMR
Training	Training_119	CRC	F	40	No	No	158	55	22.0317	No	No	Left	2.2	I	2	0	0	NA	Neg	Neg	NA	High	WT	WT	WT	pMMR
Training	Training_120	CRC	M	56	No	No	172	76.5	25.8586	No	No	Colon	3.7	I	2	0	0	Pos	Pos	Pos	NA	Intermediate	NA	NA	NA	pMMR
Training	Training_121	CRC	M	60	No	No	172	76	25.6896	Yes	No	Colon	6.5	I	2	0	0	Neg	Neg	Neg	NA	Intermediate	WT	WT	WT	pMMR
Training	Training_122	CRC	M	54	Yes	Yes	170	84	29.0657	No	No	Colon	8	I	2	0	0	NA	Neg	Neg	NA	Intermediate	WT	WT	WT	pMMR
Training	Training_123	CRC	M	63	No	No	172	57	19.2672	No	No	Colon	1.7	I	2	0	0	Pos	Neg	Neg	NA	Intermediate	WT	Mut	WT	pMMR
Training	Training_124	CRC	M	71	No	No	170	60	20.7612	Yes	No	Right	2	I	1	0	0	NA	Neg	Neg	NA	High	Mut	WT	WT	pMMR
Training	Training_125	CRC	F	48	No	No	161	59	22.7615	No	No	Colon	3	I	2	0	0	Neg	Neg	Neg	NA	Low	NA	NA	NA	NA
Training	Training_126	CRC	M	66	No	No	166	60	21.7738	No	No	Colon	6	I	2	0	0	NA	Pos	Neg	NA	Intermediate	WT	WT	WT	pMMR
Training	Training_127	CRC	F	48	No	No	160	60	23.4375	No	No	Colon	2.5	I	1	0	0	NA	Neg	Neg	NA	High	NA	NA	NA	NA
Training	Training_128	CRC	M	78	No	No	178	70	22.0932	No	Yes	Colon	4	I	2	0	0	Pos	Neg	Neg	NA	Intermediate	WT	WT	Mut	pMMR
Training	Training_129	CRC	M	64	Yes	No	170	65	22.4913	No	Yes	Left	2	I	2	0	0	NA	Pos	Neg	NA	Intermediate	WT	WT	WT	pMMR
Training	Training_130	CRC	M	53	No	No	185	65	18.992	No	No	Left	3	I	2	0	0	Pos	Neg	Neg	NA	Intermediate	WT	WT	WT	pMMR
Training	Training_131	CRC	F	56	No	No	153	55	23.4952	No	No	Colon	1.8	I	2	0	0	NA	Neg	Neg	NA	Intermediate	WT	WT	WT	pMMR
Training	Training_132	CRC	F	70	No	No	153	51	21.7865	No	Yes	Colon	2.2	I	1	0	0	Pos	Neg	Neg	NA	Intermediate	WT	Mut	WT	pMMR
Training	Training_133	CRC	F	51	No	No	168	70	24.8016	Yes	No	Colon	2	I	2	0	0	Neg	Neg	Neg	NA	Low	Mut	WT	WT	pMMR
Training	Training_134	CRC	F	52	No	No	165	87	31.9559	Yes	No	Left	1.8	I	1	0	0	Neg	Neg	Neg	NA	Intermediate	Mut	WT	WT	pMMR
Training	Training_135	CRC	F	62	No	No	162	58	22.1003	No	Yes	Colon	4.2	I	2	0	0	Pos	Neg	Neg	NA	Intermediate	WT	Mut	WT	pMMR
Training	Training_136	CRC	F	52	No	No	155	39	16.2331	No	No	Right	4	I	2	0	0	NA	Neg	Neg	NA	High	NA	NA	NA	NA
Training	Training_137	CRC	M	59	No	No	172	58	19.6052	No	No	Colon	3	I	2	0	0	NA	Neg	Neg	NA	High	WT	WT	WT	pMMR
Training	Training_138	CRC	M	71	No	No	170	49	16.955	No	No	Right	1	I	1	0	0	Neg	Neg	Neg	NA	Intermediate	NA	NA	NA	pMMR
Training	Training_139	CRC	M	61	No	No	165	63	23.1405	No	No	Colon	0.8	I	1	0	0	Neg	Neg	Neg	NA	Intermediate	WT	WT	WT	pMMR

Training	Training_140	CRC	F	56	No	No	160	60	23.4375	No	No	Right	2.4	I	2	0	0	NA	Neg	Neg	NA	Low	WT	WT	WT	dMMR
Training	Training_141	CRC	F	63	No	No	162	69	26.2917	Yes	Yes	Right	1.5	I	2	0	0	Neg	Neg	Pos	NA	Intermediate	Mut	WT	WT	pMMR
Training	Training_142	CRC	F	71	No	No	168	55	19.487	No	Yes	Colon	2.2	I	2	0	0	Neg	Neg	Neg	NA	Intermediate	WT	WT	WT	pMMR
Training	Training_143	CRC	M	61	No	No	171	74.5	25.4779	No	Yes	Colon	2.5	I	1	0	0	Pos	Neg	Neg	NA	Low	Mut	WT	WT	pMMR
Training	Training_144	CRC	F	72	No	No	164	64	23.7954	No	No	Left	2.5	I	1	0	0	Pos	Neg	Neg	NA	High	WT	WT	WT	dMMR
Training	Training_145	CRC	M	60	No	Yes	178	70	22.0932	No	No	Left	2.5	I	2	0	0	Neg	Neg	Neg	NA	High	WT	WT	WT	pMMR
Training	Training_146	CRC	F	54	No	No	161	63	24.3046	No	No	Colon	1.8	I	1	0	0	Pos	Neg	Neg	NA	High	WT	WT	WT	pMMR
Training	Training_147	CRC	M	82	No	No	158	79	31.6456	No	Yes	Colon	3	I	2	0	0	NA	Neg	Neg	NA	Low	WT	WT	Mut	pMMR
Training	Training_148	CRC	M	63	No	No	167	50	17.9282	No	No	Colon	5	I	2	0	0	Pos	Neg	Neg	NA	Intermediate	WT	WT	WT	pMMR
Training	Training_149	CRC	F	63	No	No	150	52.5	23.3333	No	No	Colon	2	I	1	0	0	Neg	Neg	Neg	NA	Intermediate	WT	WT	WT	pMMR
Training	Training_150	CRC	F	62	No	No	164	58	21.5645	No	No	Colon	1.3	I	1	0	0	Pos	Neg	Neg	NA	High	WT	WT	WT	pMMR
Training	Training_151	CRC	F	56	No	No	163	60	22.5827	No	No	Left	3	I	1	0	0	NA	Neg	Neg	NA	Intermediate	Mut	WT	WT	pMMR
Training	Training_152	CRC	F	65	No	No	165	64	23.5078	No	No	Colon	3	I	2	0	0	Pos	Neg	Neg	NA	High	WT	WT	WT	pMMR
Training	Training_153	CRC	F	77	No	No	156	50	20.5457	No	No	Colon	4	I	2	0	0	Pos	Neg	Neg	NA	Intermediate	Mut	WT	WT	pMMR
Training	Training_154	CRC	F	81	No	No	150	55	24.4444	No	Yes	Colon	3.5	I	2	0	0	Pos	Neg	Neg	NA	Intermediate	WT	WT	WT	pMMR
Training	Training_155	CRC	M	55	No	No	166	73	26.4915	Yes	No	Colon	2	I	1	0	0	Pos	Neg	Neg	NA	High	NA	NA	NA	NA
Training	Training_156	CRC	M	65	No	No	161	55	21.2183	No	No	Colon	2	I	2	0	0	Neg	Neg	Neg	NA	Intermediate	WT	WT	WT	pMMR
Training	Training_157	CRC	M	47	No	Yes	168	69	24.4473	No	No	Left	1	I	1	0	0	NA	Pos	Neg	NA	High	NA	NA	NA	NA
Training	Training_158	CRC	M	68	No	No	172	62	20.9573	No	No	Right	2.5	I	1	0	0	NA	Neg	Neg	NA	Intermediate	Mut	WT	WT	pMMR
Training	Training_159	CRC	M	57	No	No	177	87	27.7698	No	Yes	Left	6.6	I	2	0	0	Neg	Neg	Neg	NA	High	WT	WT	WT	pMMR
Training	Training_160	CRC	M	63	Yes	Yes	174	74	24.4418	No	Yes	Left	7	I	2	0	0	Pos	Neg	Neg	NA	High	WT	WT	WT	pMMR
Training	Training_161	CRC	M	48	No	No	158	44	17.6254	No	No	Right	0.8	I	1	0	0	NA	Neg	Neg	NA	High	NA	NA	NA	NA
Training	Training_162	CRC	M	70	Yes	No	169	66	23.1084	Yes	No	Colon	4	I	2	0	0	NA	Neg	Pos	NA	Intermediate	WT	WT	WT	pMMR
Training	Training_163	CRC	M	61	No	No	166	60	21.7738	No	No	Colon	5	I	1	0	0	Neg	Neg	Neg	NA	High	NA	NA	NA	NA
Training	Training_164	CRC	F	44	No	No	160	59	23.0469	No	No	Colon	2	I	2	0	0	NA	Neg	Neg	NA	Low	WT	WT	WT	pMMR
Training	Training_165	CRC	M	53	No	No	170	68.5	23.7024	No	No	Colon	2	I	1	0	0	Pos	Neg	Neg	NA	Intermediate	WT	WT	WT	pMMR
Training	Training_166	CRC	M	64	No	No	162	55	20.9572	No	Yes	Left	5.5	I	2	0	0	NA	Neg	Neg	NA	High	WT	WT	WT	pMMR
Training	Training_167	CRC	F	74	No	No	165	85	31.2213	No	No	Left	4	I	2	0	0	Neg	Neg	Pos	NA	Low	Mut	WT	WT	pMMR
Training	Training_168	CRC	M	54	No	No	172	60	20.2812	No	Yes	Colon	2.5	I	2	0	0	Neg	Neg	Neg	NA	Intermediate	WT	WT	WT	pMMR
Training	Training_169	CRC	M	54	Yes	Yes	180	93	28.7037	No	No	Colon	3	I	1	0	0	Neg	Neg	Neg	NA	High	Mut	WT	WT	pMMR
Training	Training_170	CRC	F	73	No	No	144	49	23.6304	No	Yes	Right	4	I	2	0	0	Neg	Neg	Neg	NA	Intermediate	WT	WT	Mut	dMMR
Training	Training_171	CRC	M	81	No	No	165	45	16.5289	No	No	Left	1	I	1	0	0	Neg	Neg	Neg	NA	High	WT	WT	WT	pMMR
Training	Training_172	CRC	M	60	Yes	Yes	176	80	25.8264	No	No	Colon	5	I	2	0	0	Pos	Neg	Neg	NA	Intermediate	Mut	WT	WT	pMMR
Training	Training_173	CRC	M	65	No	No	162	65	24.7676	Yes	No	Left	1.5	I	2	0	0	Neg	Neg	Neg	NA	High	NA	NA	NA	dMMR
Training	Training_174	CRC	F	68	No	No	167	74	26.5338	No	No	Left	2	I	1	0	0	NA	Neg	Neg	NA	Intermediate	NA	NA	NA	NA
Training	Training_175	CRC	M	47	No	No	175	65	21.2245	No	No	Colon	2.5	I	2	0	0	Pos	Neg	Neg	NA	Intermediate	WT	WT	WT	pMMR
Training	Training_176	CRC	F	54	No	No	160	63	24.6094	No	No	Right	1.4	I	1	0	0	Neg	Neg	Neg	NA	Intermediate	WT	WT	WT	pMMR
Training	Training_177	CRC	M	68	No	No	164	70	26.0262	No	No	Right	1.5	I	1	0	0	NA	Neg	Neg	NA	High	WT	WT	WT	pMMR
Training	Training_178	CRC	M	63	No	No	172	81	27.3797	No	No	Colon	2.8	I	2	0	0	Neg	Neg	Pos	NA	Intermediate	Mut	WT	WT	pMMR
Training	Training_179	CRC	M	43	Yes	No	170	45.5	15.7439	No	No	Left	1	0	Tis	0	0	Neg	Neg	Neg	NA	High	NA	NA	NA	NA
Training	Training_180	CRC	M	67	No	No	169	57	19.9573	No	No	Colon	3.8	0	Tis	0	0	NA	Neg	Neg	NA	High	NA	NA	NA	NA
Training	Training_181	CRC	M	79	No	No	150	45	20	No	Yes	Colon	4	0	Tis	0	0	Neg	Neg	Neg	NA	High	NA	NA	NA	NA
Training	Training_182	CRC	M	77	No	No	165	75	27.5482	Yes	Yes	Right	4.7	0	Tis	0	0	Neg	Neg	Neg	NA	High	NA	NA	NA	NA
Training	Training_183	CRC	M	63	No	No	160	51	19.9219	No	No	Colon	1	0	Tis	0	0	NA	Neg	Neg	NA	High	NA	NA	NA	NA
Training	Training_184	CRC	M	51	No	No	176	76	24.5351	No	No	Left	5.5	0	Tis	0	0	Neg	Neg	Neg	NA	High	NA	NA	NA	NA
Training	Training_185	CRC	F	68	No	No	163	71	26.7229	No	Yes	Right	1.8	0	Tis	0	0	Pos	Neg	Neg	NA	High	NA	NA	NA	NA
Training	Training_186	CRC	F	63	No	No	158	58	23.2335	No	No	Left	4	0	Tis	0	0	NA	Neg	Neg	NA	High	NA	NA	NA	NA
Training	Training_187	CRC	M	63	Yes	Yes	177	70	22.3435	No	No	Left	2	0	Tis	0	0	Pos	Neg	Neg	NA	High	NA	NA	NA	NA
Training	Training_188	CRC	M	63	Yes	Yes	170	79	27.3356	No	Yes	Colon	1.5	0	Tis	0	0	NA	Neg	Neg	NA	High	NA	NA	NA	NA
Training	Training_189	CRC	M	72	No	No	175	73	23.8367	No	No	Colon	1.7	0	Tis	0	0	Neg	Neg	Neg	NA	High	NA	NA	NA	NA
Training	Training_190	CRC	F	71	No	No	158	56	22.4323	No	No	Left	2	0	Tis	0	0	NA	Pos	Neg	NA	High	NA	NA	NA	NA
Training	Training_191	CRC	F	77	No	No	157	48.5	19.6763	No	No	Colon	2.2	0	Tis	0	0	Neg	Neg	Neg	NA	High	NA	NA	NA	NA
Training	Training_192	CRC	F	64	No	No	160	55	21.4844	No	No	Colon	5	0	Tis	0	0	NA	Neg	Neg	NA	High	NA	NA	NA	NA
Training	Training_193	CRC	M	62	No	No	160	49	19.1406	No	No	Right	3	0	Tis	0	0	Neg	Neg	Neg	NA	High	NA	NA	NA	NA
Training	Training_194	CRC	M	49	No	No	164	62	23.0518	No	No	Colon	3	0	Tis	0	0	Neg	Neg	Neg	NA	High	NA	NA	NA	NA
Training	Training_195	CRC	M	48	No	No	179	73	22.7833	No	No	Right	3.5	0	Tis	0	0	Pos	Neg	Neg	NA	High	NA	NA	NA	NA
Test	Test_001	advCRA	F	57	No	No	155	60	24.974	No	No	Colon	3	NA	NA	NA	NA	Pos	Neg	Neg	sessile	NA	NA	NA	NA	NA
Test	Test_002	advCRA	F	55	No	No	158	56.5	22.6326	No	No	Colon	3.5	NA	NA	NA	NA	Neg	Neg	Neg	sessile	Low	NA	NA	NA	NA
Test	Test_003	advCRA	F	56	No	No	153	58	24.7768	No	Yes	Colon	1.8	NA	NA	NA	NA	Neg	Neg	Neg	pedunculated	High	NA	NA	NA	NA
Test	Test_004	advCRA	F	45	No	No	152	45	19.4771	No	No	Right	4.6	NA	NA	NA	NA	Neg	Neg	Neg	sessile	High	NA	NA	NA	NA
Test	Test_005	advCRA	F	69	No	No	165	65	23.8751	Yes	No	Right	3.5	NA	NA	NA	NA	Neg	Neg	Neg	sessile	Low	NA	NA	NA	NA
Test	Test_006	advCRA	M	66	No	No	167	73	26.1752	Yes	Yes	Left	3	NA	NA	NA	NA	Pos	Pos	Pos	pedunculated	High	NA	NA	NA	NA
Test	Test_007	advCRA	F	66	No	No	156	47	19.313	No	Yes	Colon	2.5	NA	NA	NA	NA	NA	Neg	Neg	sessile	Low	NA	NA	NA	NA
Test	Test_008	advCRA	F	59	No	No	160	62	24.2188	No	No	Right	1.5	NA	NA	NA	NA	Neg	Neg	Neg	sessile	High	NA	NA	NA	NA
Test	Test_009	advCRA	F	68	No	No	159	46	18.1955	No	No	Colon	2	NA	NA	NA	NA	NA	Neg	Neg	sessile	Low	NA	NA	NA	NA
Test	Test_010	advCRA	M	69	No	No	165	60	18.726	No	No	Right	1.5	NA	NA	NA	NA	Neg	Neg	Neg	sessile	High	NA	NA	NA	NA
Test	Test_011	advCRA	F	60	No	No	155	57.5	23.9334	No	Yes	Colon	3.3	NA	NA	NA	NA	Neg	Pos	Neg	sessile	Low	NA	NA	NA	NA
Test	Test_012	advCRA	M	69	No	No	175	78	25.4694	No	No	Right	1.1	NA	NA	NA	NA	Neg	Neg	Neg	sessile	Low	NA	NA	NA	NA
Test	Test_013	advCRA	M	63	No	No	171	65	22.2291	No	No	Left	6	NA	NA	NA	NA	NA	Neg	Neg	sessile	Low	NA	NA	NA	NA
Test	Test_014	advCRA	F	63	No	No	160	50	19.5313	No	No	Colon	3	NA	NA	NA	NA	NA	Neg	Neg	sessile	Low	NA	NA	NA	NA
Test	Test_015	advCRA	F	73	No	No	158	69	27.6398	No	No	Right	6	NA	NA	NA	NA	NA	Neg	Neg	sessile	Low	NA	NA	NA	NA
Test	Test_016	advCRA	M	75	No	No	170	65	22.4913	No	No	Colon	3	NA	NA	NA	NA	NA	Neg	Neg	sessile	High	NA	NA	NA	NA
Test	Test_017	advCRA	F	54	No	No	155	54	22.4766	No	Yes	Left	4	NA	NA	NA	NA	NA	Neg	Neg	sessile	Low	NA	NA	NA	NA
Test	Test_018	advCRA	M	61	No	No	168	60	21.2585	No	No	Colon	2.7	NA	NA	NA	NA	Pos	Neg	Pos	pedunculated	Low	NA	NA	NA	NA
Test	Test_019	advCRA	M	79	Yes	Yes	160	45	17.5781	No	No	Colon	5.5	NA	NA	NA	NA	Neg	Neg	Neg	sessile	High	NA	NA	NA	NA
Test	Test_020	advCRA	M	59	No	No	170	81	28.0277	No	No	Colon	3.5	NA	NA	NA	NA	NA	Neg	Pos	sessile	High	NA	NA	NA	NA
Test	Test_021	advCRA	F	35	No	No	156	48	19.7239	No	No	Colon	2.5	NA	NA	NA	NA	Neg	Neg	Neg	pedunculated	Low	NA	NA	NA	NA
Test	Test_022	advCRA	M	56	No	No	165	62	22.7732	No	No	Right	5	NA	NA	NA	NA	NA	Neg	Neg	sessile	Low	NA	NA	NA	NA
Test	Test_023	advCRA	M	55	No	No	175	73.4	23.9673	No	Yes	Right	4	NA	NA	NA	NA	Pos	Neg	Neg	sessile	Low	NA	NA	NA	NA
Test	Test_024	advCRA	M	68	No	No	176	80	25.8264	No	No	Colon	4.5	NA	NA	NA	NA	Neg	Neg	Neg	sessile	High	NA	NA	NA	NA
Test	Test_025	advCRA	F	47	No	No	161	58	22.3757	No	No	Right	5.5	NA	NA	NA	NA	NA	Neg	Neg	pedunculated	High	NA	NA	NA	NA
Test	Test_026	advCRA	M	55	No	No	173	74	24.7252	No	Yes	Left	4	NA	NA	NA	NA	Pos	Neg	Neg	sessile	High	NA	NA	NA	NA
Test	Test_027	advCRA	F	72	No	No	155	68	28.3039	Yes	Yes	Right	2.5	NA	NA	NA	NA	Neg	Neg	Neg	pedunculated	High	NA	NA	NA	NA
Test	Test_028	advCRA	M	47	No	No	162	69	26.2917	No	Yes	Right	3.5	NA	NA	NA	NA	Pos	Neg	Neg	pedunculated	High	NA	NA	NA	NA
Test	Test_029	advCRA	F	65	No	No	164	52	19.3337	No	No	Right	4	NA	NA	NA	NA	Pos	Neg	Neg	sessile	High	NA	NA	NA	NA
Test	Test_030	advCRA	M	28	No	No	178	65	20.5151	No	No	Left	4	NA	NA	NA	NA	Pos	Neg	Neg	pedunculated	Low	NA	NA	NA	NA
Test	Test_031	advCRA	F	55	No	No	158	51	20.4294	No	No	Colon	1.2	NA	NA	NA	NA	Neg	Neg	Neg	sessile	Low	NA	NA	NA	NA
Test	Test_032	advCRA	F	54	No	No	166	60.5	21.9553	No	No	Right	7	NA	NA	NA	NA	NA	Neg	Neg	sessile	High	NA	NA	NA	NA
Test	Test_033	advCRA	M	43	No	No	181	73	22.2826	No	No	Colon	1	NA	NA	NA	NA	NA	Neg	Neg	pedunculated	High	NA	NA	NA	NA
Test	Test_034	advCRA	F	51	No	No	160	62	24.2188	No	No	Colon	5.5	NA	NA	NA	NA	Neg	Neg	Neg	sessile	High	NA	NA	NA	NA
Test	Test_035	advCRA	M	53	Yes	Yes	175	79	25.7959	No	No	Colon	6	NA	NA	NA	NA	Neg	Neg	Neg	sessile	High	NA	NA	NA	NA
Test	Test_036	advCRA	F	49	No	No	162	55	20.9572	No	No	Left	2.5	NA	NA	NA	NA	Pos	Pos	Neg	pedunculated	High	NA	NA	NA	NA
Test	Test_037	advCRA	M	66	No	No	165	60	22.0386	Yes	No	Right	8	NA	NA	NA	NA	Neg	Neg	Neg	pedunculated	Low	NA	NA	NA	NA
Test	Test_038	advCRA	F	67	No	No	156	72.5	29.7913	No	Yes	Colon	1.5	NA	NA	NA	NA	Neg	Neg	Neg	pedunculated	Low	NA	NA	NA	NA
Test	Test_039	advCRA	F	59	No	No	157	57	23.1247	Yes	Yes	Left	4.5	NA	NA	NA	NA	Pos	Pos	Neg	sessile	High	NA	NA	NA	NA
Test	Test_040	advCRA	F	49	No	No	159	56	22.151	No	No	Right	2.2	NA	NA	NA	NA	Neg	Pos	Neg	sessile	High	NA	NA	NA	NA
Test	Test_041	advCRA	M	54	No	No	175	72	23.5102	No	No	Colon	1.5	NA	NA	NA	NA	Neg	Neg	Neg	sessile	Low	NA	NA	NA	NA
Test	Test_042	advCRA	M	50	No	No	182	73	22.0384	No	No	Colon	2	NA	NA	NA	NA	Neg	Neg	Neg	sessile	Low	NA	NA	NA	NA
Test	Test_043	advCRA	F	49	No	No	160	60	23.4375	No	No	Right	2.5	NA	NA	NA	NA	NA	Neg	Neg	pedunculated	High	NA	NA	NA	NA
Test	Test_044	advCRA	M	36	No	No	168	67	23.7387	No	No	Right	3	NA	NA	NA	NA	NA	Neg	Neg	pedunculated	High	NA	NA	NA	NA
Test	Test_045	advCRA	F	70	No	No	153	58	24.7768	No	No	Colon	3	NA	NA	NA	NA	Pos	Neg	Neg	sessile	Low	NA	NA	NA	NA
Test	Test_046	advCRA	F	70	No	No	156	57.5	23.6275	No	No	Left	2.3	NA	NA	NA	NA	Neg	Neg	Neg	sessile	Low	NA	NA	NA	NA
Test	Test_047	CRC	M	41	No	Yes	172	92	31.0979	No	No	Colon	3.8	I	2	0	0	Pos	Neg	Neg	NA	Intermediate	WT	WT	WT	pMMR
Test	Test_048	CRC	M	65	No	No	172	64	21.6333	No	No	Left	2.5	I	1	0	0	Pos	Neg	Neg	NA	High	WT	WT	WT	pMMR
Test	Test_049	CRC	F	69	No	No	152	56.5	24.4546	No	Yes	Colon	3	I	2	0	0	NA	Neg	Neg	NA	Intermediate	NA	NA	NA	NA
Test	Test_050	CRC	M	77	No	No	167	67	24.0238	No	Yes	Right	2.8	I	2	0	0	Neg	Neg	Neg	NA	High	Mut	WT	WT	pMMR
Test	Test_051	CRC	M	76	No	No	170	60.5	20.9343	No	Yes	Colon	5.5	I	2	0	0	Neg	Neg	Neg	NA	Intermediate	WT	WT	WT	pMMR
Test	Test_052	CRC	M	43	No	No	165	66	24.2424	No	No	Colon	3	I	1	0	0	Pos	Neg	Neg	NA	High	WT	Mut	WT	pMMR
Test	Test_053	CRC	M	58	No	No	168	63	22.3214	No	No	Colon	3	I	2	0	0	Neg	Neg	Neg	NA	Intermediate	WT	WT	WT	pMMR
Test	Test_054	CRC	M	81	No	No	169	62	21.7079	No	No	Left	1	I	1	0	0	NA	Pos	Neg	NA	High	NA	NA	NA	pMMR
Test	Test_055	CRC	M	73	No	No	169	75	26.2596	No	Yes	Colon	2.5	I	2	0	0	Neg	Neg	Neg	NA	Low	Mut	WT	WT	pMMR
Test	Test_056	CRC	F	67	No	No	160	55.5	21.6797	No	No	Colon	2.5	I	2	0	0	Neg	Neg	Neg	NA	Intermediate	Mut	WT	WT	pMMR
Test	Test_057	CRC	M	66	No	No	170	68	23.5294	No	No	Colon	2.5	I	2	0	0	Neg	Neg	Neg	NA	Intermediate	WT	WT	WT	pMMR
Test	Test_058	CRC	F	59	No	No	158	62	24.8358	No	No	Left	2.5	I	2	0	0	NA	Pos	Neg	NA	Intermediate	Mut	WT	WT	pMMR
Test	Test_059	CRC	F	71	No	No	157	46	18.662	No	No	Right	1.5	I	2	0	0	Pos	Neg	Neg	NA	Intermediate	WT	WT	Mut	pMMR
Test	Test_060	CRC	M	59	No	No	174	90	29.7265	Yes	No	Colon	4.4	I	2	0	0	NA	Neg	Neg	NA	Intermediate	Mut	WT	WT	pMMR
Test	Test_061	CRC	M	79	No	No	168	52	18.424	No	No	Left	4	I	2	0	0	Pos	Neg	Neg	NA	Intermediate	WT	WT	WT	pMMR
Test	Test_062	CRC	M	74	No	No	169	75	26.2596	No	No	Left	2.3	I	2	0	0	NA	Neg	Neg	NA	Low	WT	WT	WT	pMMR
Test	Test_063	CRC	M	67	No	No	178	70	22.0932	No	No	Left	5.5	I	2	0	0	Neg	Neg	Neg	NA	Intermediate	WT	WT	WT	pMMR
Test	Test_064	CRC	M	66	No	No	170	74	25.6055	No	No	Colon	4	I	2	0	0	Neg	Neg	Neg	NA	Intermediate	WT	WT	WT	pMMR
Test	Test_065	CRC	M	69	No	No	167	72	25.8166	No	Yes	Right	3.5	I	2	0	0	Pos	Neg	Neg	NA	Intermediate	Mut	WT	WT	pMMR
Test	Test_066	CRC	M	75	No	No	168	67	23.7387	No	No	Colon	4.5	I	2	0	0	Neg	Neg	Neg	NA	Intermediate	WT	WT	WT	pMMR
Test	Test_067	CRC	F	81	No	No	147	67	31.0056	No	Yes	Left	2.5	I	1	0	0	Pos	Neg	Neg	NA	Low	WT	WT	WT	pMMR
Test	Test_068	CRC	F	54	No	No	160	51	19.9219	No	No	Right	1.5	I	2	0	0	Neg	Neg	Neg	NA	Low	WT	Mut	Mut	pMMR
Test	Test_069	CRC	F	73	No	No	145	51	24.2568	Yes	Yes	Colon	2.8	I	1	0	0	NA	Pos	Pos	NA	Intermediate	NA	NA	NA	NA
Test	Test_070	CRC	M	55	Yes	Yes	172	68	22.9854	No	No	Left	2	I	1	0	0	Pos	Neg	Neg	NA	Intermediate	WT	WT	WT	pMMR
Test	Test_071	CRC	M	69	No	No	168	77.5	27.4589	No	No	Colon	5.5	I	2	0	0	Pos	Neg	Neg	NA	High	WT	WT	WT	pMMR
Test	Test_072	CRC	M	64	No	No	178	89	28.0899	No	No	Colon	3.5	I	2	0	0	NA	Neg	Neg	NA	Intermediate	Mut	WT	WT	pMMR
Test	Test_073	CRC	F	59	No	No	160	81	31.6406	Yes	Yes	Colon	2.5	I	2	0	0	NA	Neg	Neg	NA	Intermediate	WT	WT	WT	pMMR
Test	Test_074	CRC	M	57	Yes	Yes	172	92	31.0979	Yes	Yes	Left	1.5	I	1	0	0	Neg	Neg	Neg	NA	Intermediate	WT	WT	Mut	dMMR
Test	Test_075	CRC	M	67	No	No	160	79	30.8594	No	Yes	Colon	2.5	I	2	0	0	Neg	Neg	Neg	NA	Intermediate	Mut	WT	WT	pMMR
Test	Test_076	CRC	M	72	No	No	170	59	20.4152	No	No	Left	7	I	2	0	0	Pos	Neg	Pos	NA	High	WT	WT	WT	pMMR
Test	Test_077	CRC	M	46	No	No	170	71	24.5675	No	Yes	Left	1.2	I	1	0	0	NA	Neg	Neg	NA	High	WT	WT	WT	pMMR
Test	Test_078	CRC	F	54	No	No	160	58	22.6563	No	No	Colon	2.2	I	1	0	0	Pos	Neg	Neg	NA	Intermediate	WT	WT	WT	pMMR
Test	Test_079	CRC	F	55	No	No	160	58	22.6563	No	Yes	Right	1.8	I	1	0	0	Neg	Neg	Neg	NA	Intermediate	NA	NA	NA	NA
Test	Test_080	CRC	F	53	No	No	160	58	22.6563	Yes	No	Colon	2.5	I	1	0	0	Pos	Neg	Neg	NA	High	Mut	WT	WT	pMMR
Test	Test_081	CRC	M	55	No	No	158	70	28.0404	No	Yes	Colon	2	I	1	0	0	NA	Neg	Neg	NA	High	WT	WT	WT	pMMR
Test	Test_082	CRC	M	40	No	No	170	64	22.1453	No	No	Colon	2.6	I	2	0	0	NA	Neg	Neg	NA	Intermediate	Mut	WT	WT	pMMR
Test	Test_083	CRC	M	75	No	No	160	45	17.5781	No	Yes	Colon	3	I	2	0	0	Pos	Neg	Pos	NA	Intermediate	Mut	WT	WT	pMMR
Test	Test_084	CRC	M	54	No	Yes	169	75	26.2596	Yes	No	Colon	1.5	I	2	0	0	NA	Neg	Neg	NA	Intermediate	Mut	WT	WT	pMMR
Test	Test_085	CRC	M	47	No	No	167	76	27.2509	No	Yes	Left	1.3	I	1	0	0	Neg	Neg	Neg	NA	Intermediate	NA	NA	NA	pMMR
Test	Test_086	CRC	M	64	No	No	165	70	25.7117	No	Yes	Colon	2.8	I	2	0	0	Pos	Neg	Neg	NA	Intermediate	Mut	WT	WT	pMMR

Test	Test_087	CRC	M	47	No	No	167	76	27.2509	No	No	Colon	4	I	2	0	0	NA	Neg	Neg	NA	Low	WT	WT	WT	dMMR
Test	Test_088	CRC	F	69	No	No	154	58	24.4561	No	Yes	Right	2.8	I	2	0	0	Pos	Neg	Neg	NA	Intermediate	NA	NA	NA	NA
Test	Test_089	CRC	F	73	No	No	160	62	24.2188	No	No	Colon	1.8	I	1	0	0	NA	Neg	Neg	NA	Intermediate	Mut	WT	WT	pMMR
Test	Test_090	CRC	F	61	No	No	160	58	22.6563	Yes	Yes	Colon	3.2	I	2	0	0	NA	Pos	Neg	NA	Low	Mut	WT	WT	pMMR
Test	Test_091	CRC	M	66	No	No	160	80	31.25	No	No	Right	6	I	2	0	0	NA	Neg	Neg	NA	Intermediate	WT	Mut	WT	pMMR
Test	Test_092	CRC	M	55	No	No	170	69	23.8754	No	Yes	Left	2.3	I	1	0	0	Pos	Neg	Neg	NA	Intermediate	WT	WT	WT	pMMR
Test	Test_093	CRC	M	64	No	No	175	61	19.9184	No	No	Left	5.5	I	2	0	0	Pos	Neg	Neg	NA	Intermediate	Mut	WT	WT	pMMR
Test	Test_094	CRC	M	57	No	No	160	62	24.2188	No	No	Colon	1.8	I	1	0	0	Pos	Neg	Neg	NA	Intermediate	WT	WT	WT	pMMR
Test	Test_095	CRC	M	50	No	No	170	70	24.2215	No	No	Colon	2.2	I	1	0	0	NA	Neg	Neg	NA	Intermediate	WT	WT	Mut	pMMR
Test	Test_096	CRC	M	56	No	No	170	56	19.3772	No	No	Colon	2	I	2	0	0	Neg	Neg	Neg	NA	Intermediate	WT	WT	WT	pMMR
Test	Test_097	CRC	M	71	No	No	177	50.5	16.1193	No	No	Left	3.2	I	1	0	0	NA	Neg	Neg	NA	Intermediate	WT	WT	WT	dMMR
Test	Test_098	CRC	M	51	No	No	174	65	21.4692	No	No	Colon	1.8	I	2	0	0	Pos	Neg	Neg	NA	High	Mut	WT	WT	NA
Test	Test_099	CRC	M	71	No	No	169	48	27.31	Yes	Yes	Right	3	I	2	0	0	Neg	Neg	Neg	NA	Low	WT	WT	WT	pMMR
Test	Test_100	CRC	M	64	No	No	174	75	24.7721	No	No	Colon	2.5	I	2	0	0	Neg	Neg	Neg	NA	Intermediate	WT	WT	WT	pMMR
Test	Test_101	CRC	M	74	No	No	165	61	22.4059	No	Yes	Colon	4	I	2	0	0	NA	Neg	Neg	NA	Intermediate	Mut	WT	WT	pMMR
Test	Test_102	CRC	M	50	No	No	180	90	27.7778	No	No	Colon	3	I	1	0	0	NA	Neg	Pos	NA	High	Mut	WT	WT	pMMR
Test	Test_103	CRC	M	62	No	No	170	60	20.7612	No	No	Colon	4	I	2	0	0	Pos	Neg	Neg	NA	Intermediate	WT	WT	WT	pMMR
Test	Test_104	CRC	M	55	No	No	180	93	28.7037	No	No	Left	4.5	I	2	0	0	Pos	Neg	Neg	NA	Intermediate	WT	WT	WT	pMMR
Test	Test_105	CRC	M	67	No	No	176	83	26.7949	Yes	Yes	Colon	6.5	I	2	0	0	NA	Neg	Neg	NA	Low	Mut	WT	WT	pMMR
Test	Test_106	CRC	F	71	No	No	162	70	26.6728	No	Yes	Left	3.5	I	2	0	0	NA	Neg	Neg	NA	Intermediate	WT	WT	WT	pMMR
Test	Test_107	CRC	F	62	No	No	162	61.5	23.4339	No	No	Left	6	I	2	0	0	Pos	Neg	Neg	NA	High	WT	WT	WT	pMMR
Test	Test_108	CRC	F	71	No	No	156	44	18.0802	No	No	Right	3.5	I	2	0	0	NA	Neg	Neg	NA	Intermediate	Mut	WT	WT	pMMR
Test	Test_109	CRC	M	60	No	No	171	65	22.2291	No	No	Colon	3.5	I	1	0	0	Pos	Neg	Neg	NA	Intermediate	Mut	WT	WT	pMMR
Test	Test_110	CRC	M	45	No	No	160	65	25.3906	No	Yes	Right	2	I	2	0	0	Neg	Neg	Neg	NA	High	Mut	WT	WT	dMMR
Test	Test_111	CRC	F	50	No	No	165	47	17.2635	No	No	Colon	3.5	I	2	0	0	Pos	Neg	Neg	NA	Intermediate	WT	WT	WT	pMMR
Test	Test_112	CRC	M	55	No	Yes	169	70	24.5089	No	No	Colon	1.5	I	1	0	0	Neg	Neg	Neg	NA	High	WT	WT	WT	pMMR
Test	Test_113	CRC	M	62	No	No	173	53.5	17.8756	No	No	Right	3.5	0	Tis	0	0	NA	Pos	Pos	NA	High	NA	NA	NA	NA
Test	Test_114	CRC	F	68	No	No	165	70	25.7117	No	Yes	Left	4	0	Tis	0	0	Pos	Neg	Neg	NA	High	NA	NA	NA	NA
Test	Test_115	CRC	M	69	No	No	170	87	30.1038	No	No	Left	1.3	0	Tis	0	0	NA	Neg	Neg	NA	High	NA	NA	NA	NA
Test	Test_116	CRC	F	80	No	No	155	59.5	24.7659	No	Yes	Colon	8	0	Tis	0	0	Pos	Neg	Neg	NA	High	NA	NA	NA	NA
Test	Test_117	CRC	F	68	No	No	158	75	30.0433	No	Yes	Colon	2.5	0	Tis	0	0	NA	Neg	Neg	NA	High	NA	NA	NA	NA
Test	Test_118	CRC	M	52	No	No	170	62.5	21.6263	No	No	Colon	6	0	Tis	0	0	Pos	Neg	Pos	NA	High	NA	NA	NA	NA
Test	Test_119	CRC	F	68	No	No	160	60	23.4375	No	No	Colon	4.5	0	Tis	0	0	NA	Neg	Neg	NA	High	NA	NA	NA	NA
Test	Test_120	CRC	M	67	No	No	169	80	28.0102	No	Yes	Colon	3	0	Tis	0	0	Neg	Neg	Pos	NA	High	NA	NA	NA	NA
Test	Test_121	CRC	F	62	No	No	157	75	30.4272	No	No	Colon	2.7	I	2	0	0	NA	Neg	Neg	NA	Intermediate	WT	Mut	WT	pMMR
Test	Test_122	CRC	F	56	No	No	158	52	20.83	No	No	Colon	3	I	2	0	0	NA	Neg	Pos	NA	Intermediate	Mut	WT	WT	pMMR
Test	Test_123	CRC	F	49	No	No	160	59	23.0469	No	No	Left	2	I	1	0	0	Pos	Neg	Neg	NA	Intermediate	WT	WT	WT	pMMR
Test	Test_124	CRC	F	64	No	No	156	63.5	26.093	No	No	Colon	3.2	I	2	0	0	Pos	Pos	Neg	NA	Intermediate	WT	WT	WT	pMMR
Test	Test_125	CRC	M	63	No	No	172	90	30.4218	No	No	Right	2.2	I	2	0	0	Neg	Neg	Pos	NA	Intermediate	WT	WT	WT	pMMR
Test	Test_126	CRC	F	58	No	No	160	55	21.4844	No	No	Right	1.8	I	1	0	0	Neg	Neg	Neg	NA	Intermediate	NA	NA	NA	pMMR
Test	Test_127	CRC	M	67	No	No	165	62	22.7732	No	Yes	Right	2.5	I	2	0	0	Neg	Neg	Neg	NA	Intermediate	WT	WT	WT	pMMR
Test	Test_128	CRC	F	67	No	No	165	53	19.4674	No	No	Colon	1.7	I	1	0	0	Neg	Neg	Neg	NA	Low	WT	WT	WT	pMMR
Test	Test_129	CRC	F	50	No	No	157	53	21.5019	No	No	Colon	2	I	1	0	0	NA	Neg	Pos	NA	High	Mut	WT	WT	pMMR
Test	Test_130	CRC	F	69	No	No	159	60	23.7332	No	No	Left	4	I	2	0	0	NA	Neg	Neg	NA	Intermediate	WT	WT	WT	pMMR
Test	Test_131	CRC	M	78	No	No	168	50.5	17.8926	No	No	Right	6	I	2	0	0	Pos	Neg	Neg	NA	Intermediate	WT	Mut	WT	dMMR
Test	Test_132	CRC	F	67	No	No	158	65	26.0375	No	No	Right	1.3	I	1	0	0	Pos	Neg	Neg	NA	Intermediate	WT	WT	WT	pMMR
Test	Test_133	CRC	M	73	No	No	165	70	25.7117	Yes	No	Colon	3.5	I	1	0	0	Neg	Neg	Neg	NA	Intermediate	Mut	WT	WT	pMMR
Test	Test_134	CRC	F	63	No	No	155	52	21.6441	No	No	Right	2	I	2	0	0	NA	Neg	Neg	NA	Intermediate	Mut	WT	WT	pMMR
Test	Test_135	CRC	M	65	No	No	168	70	24.8016	No	No	Colon	2	I	2	0	0	NA	Neg	Neg	NA	Intermediate	WT	Mut	WT	pMMR
Test	Test_136	CRC	M	75	No	No	172	70	23.6614	No	No	Colon	4.5	I	2	0	0	NA	Pos	Pos	NA	Intermediate	Mut	WT	WT	pMMR
Test	Test_137	CRC	F	57	No	No	160	67.5	26.3672	No	Yes	Colon	4.8	I	2	0	0	Pos	Neg	Neg	NA	Intermediate	Mut	WT	WT	pMMR
Test	Test_138	CRC	M	59	No	No	176	90	29.0548	No	No	Colon	2.5	I	1	0	0	Neg	Neg	Pos	NA	High	NA	NA	NA	NA
Test	Test_139	CRC	F	71	No	No	156	58	23.833	No	No	Colon	4.5	I	2	0	0	Pos	Pos	Pos	NA	Low	Mut	WT	WT	pMMR
Test	Test_140	CRC	M	67	No	No	168	85	30.1162	No	Yes	Colon	3	I	2	0	0	NA	Neg	Neg	NA	Intermediate	Mut	WT	WT	pMMR
Test	Test_141	CRC	M	59	No	No	160	75	29.2969	No	Yes	Colon	4	I	2	0	0	Pos	Neg	Neg	NA	Intermediate	WT	WT	WT	pMMR
Test	Test_142	CRC	F	73	No	No	165	60	22.0386	No	No	Colon	3	I	2	0	0	Pos	Neg	Neg	NA	Intermediate	Mut	WT	WT	pMMR
Test	Test_143	CRC	M	68	Yes	No	177	80	25.5354	No	No	Right	1.3	I	1	0	0	NA	Pos	Pos	NA	High	WT	WT	WT	pMMR
Test	Test_144	CRC	M	62	No	No	168	77.5	27.4589	Yes	Yes	Left	2	I	2	0	0	Neg	Neg	Neg	NA	High	Mut	WT	WT	pMMR
Test	Test_145	CRC	M	69	No	No	167	45	16.1354	No	No	Left	6	I	2	0	0	Pos	Neg	Neg	NA	High	WT	WT	WT	pMMR
Test	Test_146	CRC	M	57	No	No	174	60	19.8177	No	No	Colon	2	I	2	0	0	Neg	Neg	Neg	NA	Low	WT	WT	WT	pMMR
Test	Test_147	CRC	F	72	No	No	152	55	23.8054	No	No	Left	3.5	I	2	0	0	NA	Neg	Pos	NA	Intermediate	WT	WT	WT	pMMR
Test	Test_148	CRC	F	41	No	No	165	64	23.5078	No	No	Left	4	I	2	0	0	Pos	Neg	Neg	NA	Low	Mut	WT	WT	dMMR
Test	Test_149	CRC	M	54	No	No	170	72	24.9135	No	Yes	Colon	3.5	I	2	0	0	NA	Neg	Neg	NA	Intermediate	WT	WT	WT	pMMR
Test	Test_150	CRC	M	67	Yes	Yes	174	70	23.1206	No	No	Colon	1.8	I	1	0	0	Neg	Neg	Neg	NA	High	WT	WT	WT	pMMR
Test	Test_151	CRC	F	71	No	No	160	61	23.8281	No	Yes	Colon	4	I	1	0	0	Neg	Neg	Neg	NA	High	Mut	WT	WT	pMMR
Test	Test_152	CRC	F	62	No	No	148	49	22.3703	Yes	No	Right	1.4	I	1	0	0	Neg	Neg	Neg	NA	Intermediate	NA	NA	NA	pMMR
Test	Test_153	CRC	F	71	No	No	150	59	26.2222	No	No	Colon	2.5	I	1	0	0	Neg	Neg	Neg	NA	Intermediate	WT	WT	WT	pMMR
Test	Test_154	CRC	M	62	No	No	172	76	25.6896	No	No	Left	5.5	I	2	0	0	Pos	Neg	Neg	NA	Intermediate	WT	WT	WT	pMMR
Test	Test_155	CRC	F	63	No	No	153	61.5	26.2719	No	No	Right	4.5	I	2	0	0	Neg	Neg	Neg	NA	Intermediate	NA	NA	NA	NA
Test	Test_156	CRC	F	53	No	No	152	55	23.8054	No	No	Colon	2	I	1	0	0	Neg	Neg	Neg	NA	Intermediate	WT	WT	WT	pMMR
Test	Test_157	CRC	F	57	No	No	162	70	26.6728	No	No	Colon	5	I	1	0	0	Pos	Neg	Neg	NA	High	NA	NA	NA	NA
Test	Test_158	CRC	F	53	No	No	164	56	20.8209	No	No	Colon	4.5	I	1	0	0	Neg	Neg	Neg	NA	High	Mut	WT	WT	pMMR
Test	Test_159	CRC	F	63	No	No	158	65	26.0375	No	No	Colon	3	I	2	0	0	Pos	Neg	Neg	NA	Low	WT	WT	WT	pMMR
Test	Test_160	CRC	F	59	No	No	160	63	24.6094	No	No	Colon	2.5	I	2	0	0	Pos	Neg	Neg	NA	Intermediate	WT	WT	WT	pMMR
Test	Test_161	CRC	F	62	No	No	158	50	20.0288	No	Yes	Left	2.5	I	1	0	0	NA	Neg	Neg	NA	Intermediate	WT	WT	WT	pMMR
Test	Test_162	CRC	M	52	No	No	170	72	24.9135	No	No	Colon	2.3	I	2	0	0	NA	Neg	Neg	NA	Intermediate	Mut	WT	WT	pMMR
Test	Test_163	CRC	F	55	No	No	161	70.5	27.198	No	Yes	Colon	4	I	2	0	0	Pos	Neg	Pos	NA	High	Mut	WT	WT	pMMR
Test	Test_164	CRC	M	56	No	No	176	74	23.8895	No	Yes	Colon	2.5	I	2	0	0	NA	Pos	Pos	NA	Intermediate	Mut	WT	WT	pMMR
Test	Test_165	CRC	F	41	No	No	164	83	30.8596	No	No	Colon	2.5	I	1	0	0	NA	Neg	Neg	NA	High	NA	NA	NA	NA
Test	Test_166	CRC	M	73	No	No	163	65.5	24.6528	No	No	Colon	2.6	I	2	0	0	Pos	Pos	Neg	NA	Intermediate	Mut	WT	WT	dMMR
Test	Test_167	CRC	F	74	No	No	155	61	25.3902	No	Yes	Right	1.7	I	1	0	0	NA	Neg	Neg	NA	Intermediate	WT	WT	Mut	dMMR
Test	Test_168	CRC	M	54	No	No	172	83	28.0557	No	No	Left	4.8	I	2	0	0	NA	Neg	Neg	NA	Intermediate	WT	WT	WT	pMMR
Test	Test_169	CRC	M	71	No	No	160	62	24.2188	No	No	Colon	4	I	2	0	0	Pos	Pos	Pos	NA	High	Mut	WT	WT	pMMR
Test	Test_170	CRC	M	64	No	No	162	60	22.8624	No	Yes	Colon	2.5	I	2	0	0	NA	Neg	Neg	NA	Intermediate	Mut	WT	WT	pMMR
Test	Test_171	CRC	F	68	No	No	162	70	26.6728	Yes	Yes	Left	2	I	2	0	0	Pos	Neg	Neg	NA	Intermediate	Mut	WT	WT	pMMR
Test	Test_172	CRC	F	52	No	No	153	60	25.6312	No	Yes	Colon	4.2	I	2	0	0	NA	Pos	Neg	NA	Intermediate	Mut	WT	WT	dMMR
Test	Test_173	CRC	M	67	No	No	167	62	22.231	No	No	Colon	1.8	I	2	0	0	Pos	Neg	Pos	NA	Intermediate	WT	WT	WT	pMMR
Test	Test_174	CRC	M	55	No	No	170	70	24.2215	No	No	Colon	1.5	I	1	0	0	Pos	Neg	Neg	NA	Intermediate	WT	WT	WT	pMMR
Test	Test_175	CRC	M	50	Yes	No	173	64	21.3839	No	No	Colon	5	I	1	0	0	NA	Neg	Pos	NA	High	NA	NA	NA	NA
Test	Test_176	CRC	F	59	No	No	160	62	24.2188	No	Yes	Left	2.5	I	2	0	0	Neg	Neg	Neg	NA	Intermediate	WT	WT	WT	pMMR
Test	Test_177	CRC	F	67	No	No	158	60	24.0346	Yes	Yes	Left	1.5	I	1	0	0	Pos	Neg	Neg	NA	Intermediate	Mut	WT	WT	pMMR
Test	Test_178	CRC	F	61	No	No	155	65	27.0552	No	Yes	Right	3.5	I	2	0	0	NA	Neg	Neg	NA	Low	Mut	WT	WT	pMMR
Test	Test_179	CRC	M	65	No	No	164	68	25.2826	No	Yes	Left	7.5	I	2	0	0	NA	Neg	Pos	NA	Intermediate	Mut	WT	WT	pMMR
Test	Test_180	CRC	M	78	No	No	169	72	25.2092	No	No	Colon	3	I	2	0	0	Neg	Neg	Pos	NA	Intermediate	Mut	WT	WT	pMMR
Test	Test_181	CRC	M	54	No	No	163	62.5	23.5237	No	No	Right	3	I	2	0	0	Neg	Neg	Pos	NA	Low	WT	WT	Mut	pMMR
Test	Test_182	CRC	F	67	No	No	152	57.5	24.8875	No	Yes	Left	2.7	I	2	0	0	Pos	Neg	Pos	NA	Intermediate	Mut	WT	WT	pMMR
Test	Test_183	CRC	M	55	No	No	172	58	19.6052	No	No	Colon	2	I	1	0	0	Neg	Neg	Neg	NA	Intermediate	WT	WT	WT	pMMR
Test	Test_184	CRC	M	81	No	No	170	61	21.1073	No	No	Left	4.5	I	2	0	0	Pos	Neg	Neg	NA	Intermediate	WT	WT	WT	pMMR
Test	Test_185	CRC	M	47	No	No	169	61	21.3578	No	No	Colon	5	I	2	0	0	NA	Neg	Neg	NA	Intermediate	Mut	WT	WT	pMMR
Test	Test_186	CRC	F	58	No	No	153	44	18.7962	No	No	Colon	3	I	2	0	0	NA	Neg	Neg	NA	Intermediate	Mut	WT	WT	pMMR
Test	Test_187	CRC	F	63	No	No	158	58	23.2335	No	No	Left	4	0	Tis	0	0	NA	Neg	Neg	NA	High	NA	NA	NA	NA
Test	Test_188	CRC	F	63	No	No	162	53	20.1951	No	No	Right	4	0	Tis	0	0	Pos	Neg	Neg	NA	High	NA	NA	NA	NA
Test	Test_189	CRC	M	19	No	No	173	70	23.3887	No	No	Colon	2	0	Tis	0	0	NA	Neg	Neg	NA	High	NA	NA	NA	NA
Test	Test_190	CRC	F	51	No	No	163	51	19.1953	No	No	Right	1.2	0	Tis	0	0	NA	Neg	Neg	NA	High	NA	NA	NA	NA
Test	Test_191	CRC	F	69	No	No	167	55.5	19.9003	No	No	Right	3	0	Tis	0	0	NA	Neg	Neg	NA	High	NA	NA	NA	NA
Test	Test_192	CRC	M	57	No	No	160	60	23.4375	No	No	Colon	4	0	Tis	0	0	Neg	Neg	Neg	NA	High	NA	NA	NA	NA
Test	Test_193	CRC	F	65	No	No	165	58	21.3039	No	No	Colon	1.5	0	Tis	0	0	Neg	Neg	Neg	NA	High	NA	NA	NA	NA
Test	Test_194	CRC	M	62	No	No	170	68	23.5294	No	No	Left	2	0	Tis	0	0	Neg	Neg	Neg	NA	High	NA	NA	NA	NA
Test	Test_195	CRC	M	59	No	No	173	80	26.7299	Yes	Yes	Left	2.5	0	Tis	0	0	Neg	Neg	Neg	NA	High	NA	NA	NA	NA

Table S5. Evaluating 4X coverage-unified model performances in the test dataset at 90% and 98% specificities

Healthy vs advCRA + CRC							
90% specificity		Actual		98% specificity		Actual	
		advCRA/CRC	Healthy			advCRA/CRC	Healthy
Predict	advCRA/CRC	192	12	Predict	advCRA/CRC	173	2
	Healthy	3	104		Healthy	22	114
Sensitivity (95% CI)		98.5% (95.6-99.7%)		Sensitivity (95% CI)		88.7% (83.4-92.8%)	
Specificity (95% CI)		89.7% (82.6-94.5%)		Specificity (95% CI)		98.3% (93.9-99.8%)	
PPV (95% CI)		94.1% (90-96.9%)		PPV (95% CI)		98.9% (95.9-99.9%)	
NPV (95% CI)		97.2% (92-99.4%)		NPV (95% CI)		83.8% (76.5-89.6%)	
Accuracy (95% CI)		95.2% (92.2-97.3%)		Accuracy (95% CI)		92.3% (88.7-95%)	
Healthy vs advCRA							
90% specificity		Actual		98% specificity		Actual	
		advCRA	Healthy			advCRA	Healthy
Predict	advCRA	45	12	Predict	advCRA	37	2
	Healthy	1	104		Healthy	9	114
Sensitivity (95% CI)		97.8% (88.5-99.9%)		Sensitivity (95% CI)		80.4% (66.1-90.6%)	
Specificity (95% CI)		89.7% (82.6-94.5%)		Specificity (95% CI)		98.3% (93.9-99.8%)	
PPV (95% CI)		78.9% (66.1-88.6%)		PPV (95% CI)		94.9% (82.7-99.4%)	
NPV (95% CI)		99.0% (94.8-100%)		NPV (95% CI)		92.7% (86.6-96.6%)	
Accuracy (95% CI)		92.0% (86.7-95.7%)		Accuracy (95% CI)		93.2% (88.2-96.6%)	
Healthy vs CRC							
90% specificity		Actual		98% specificity		Actual	
		CRC	Healthy			CRC	Healthy
Predict	CRC	147	12	Predict	CRC	136	2
	Healthy	2	104		Healthy	13	114
Sensitivity (95% CI)		98.7% (95.2-99.8%)		Sensitivity (95% CI)		91.3% (85.5-95.3%)	
Specificity (95% CI)		89.7% (82.6-94.5%)		Specificity (95% CI)		98.3% (93.9-99.8%)	
PPV (95% CI)		92.5% (87.2-96%)		PPV (95% CI)		94.3% (90.8-96.8%)	
NPV (95% CI)		98.1% (93.4-99.8%)		NPV (95% CI)		98.6% (94.9-99.8%)	
Accuracy (95% CI)		94.3% (90.8-96.8%)		Accuracy (95% CI)		89.8% (83.1-94.4%)	

`

Table S6. Limit of detection using downsample data in the test dataset.

Healthy vs advCRA/CRC	Sensitivity at different specificities		
	**89.7% specificity**	**94.8% specificity**	**98.3% specificity**
4X coverage	98.5% (95.6-99.7%)	97.4% (94.1-99.2%)	88.7% (83.4-92.8%)
3X coverage	97.9% (94.8-99.4%)	96.9% (93.4-98.9%)	82.1% (75.9-87.2%)
2X coverage	97.9% (94.8-99.4%)	95.9% (92.1-98.2%)	84.1% (78.2-88.9%)
1X coverage	96.9% (93.4-98.9%)	94.4% (90.1-97.2%)	86.2% (80.5-90.7%)
0.5X coverage	95.4% (91.4-97.9%)	90.8% (85.8-94.4%)	85.1% (79.3-89.8%)

Table S7. Model performances for subgroups patients in the test dataset.

Subgroups		Number	Sensitivity (95% CI)*
CRC	**Stage TNM**		
	0	17	94.1% (71.3-99.9%)
	I	132	98.5% (94.6-99.8%)
	**FOBT**		
	Pos	51	100% (93.0-100%)
	Neg	43	100% (91.8-100%)
	NA	55	94.5% (84.9- 98.9%)
	**CEA**		
	Pos	24	100.0% (85.8-100.0%)
	Neg	125	97.6% (93.1- 99.5%)
	**CA199**		
	Pos	13	100.0% (75.3-100.0%)
	Neg	136	97.8% (93.7- 99.5%)
	**Size (cm)**		
	< 3	75	97.3% (90.7- 99.7%)
	> 3	74	98.6% (92.7-100.0%)
	**Grade**		
	High	44	97.7% (88.0-99.9%)
	Intermediate	90	98.9% (94.0-100.0%)
	Low	15	93.3% (68.1-99.8%)
	**MMR**		
	dMMR	9	100.0% (66.4-100.0%)
	pMMR	113	98.2% (93.8- 99.8%)
	NA	27	96.3% (81.0- 99.9%)
	**KRAS**		
	Mut	51	100.0% (93.0-100.0%)
	WT	68	97.1% (89.8- 99.6%)
	NA	30	96.7% (82.8- 99.9%)
	**NRAS**		
	Mut	6	83.3% (35.9- 99.6%)
	WT	113	99.1% (95.2-100.0%)
	NA	30	96.7% (82.8- 99.9%)
	**BRAF**		
	Mut	6	100.0% (54.1-100.0%)
	WT	113	98.2% (93.8- 99.8%)
	NA	30	96.7% (82.8- 99.9%)
	**Location**		
	Left	38	94.7% (82.3- 99.4%)
	Colon	85	98.8% (93.6-100.0%)
	Right	26	100.0% (86.8-100.0%)
	**Vascular invasion**		
	Pos	10	100.0% (69.2-100.0%)
	Neg	122	98.4% (94.2- 99.8%)
	NA	17	94.1% (71.3- 99.9%)
	**FOBT**		

advCRA	Pos	11	100.0% (71.5-100.0%)
	Neg	21	95.2% (76.2-99.9%)
	NA	14	92.9% (66.1-99.8%)
	**CEA**		
	Pos	3	100% (29.2-100.0%)
	Neg	43	95.3% (84.2- 99.4%)
	**CA199**		
	Pos	5	100.0% (47.8-100.0%)
	Neg	41	95.1% (83.5- 99.4%)
	**Size (cm)**		
	< 3	18	94.4% (72.7-99.9%)
	> 3	28	96.4% (81.7-99.9%)
	**Grade**		
	High	23	91.3% (72.0- 98.9%)
	Low	22	100.0% (84.6-100.0%)
	NA	1	100.0% (2.5-100.0%)
	**Endoscopic**		
	pedunculated	14	92.9% (66.1-99.8%)
	sessile	32	96.9% (83.8-99.9%)
	**Location**		
	Left	8	100.0% (63.1-100.0%)
	Colon	21	100.0% (83.9-100.0%)
	Right	17	88.2% (63.6- 98.5%)

***: at 94.8% specificity**
